# Effects of Dietary Protein and Lipid Levels in Practical Formulation on Growth, Feed Utilization, Body Composition, and Serum Biochemical Parameters of Growing Rockfish *Sebastes schlegeli*

**DOI:** 10.1155/2023/9970252

**Published:** 2023-08-08

**Authors:** Peiyu Li, Zhidong Song, Long Huang, Yongzhi Sun, Yuming Sun, Xiaoyan Wang, Lu Li

**Affiliations:** ^1^Yantai Key Laboratory of Quality and Safety Control and Deep Processing of Marine Food, Shandong Marine Resource and Environment Research Institute, Yantai 264006, China; ^2^Yantai Zhulin Human Resources Service Co. Ltd, Yantai 264006, China; ^3^Shandong Shengsuo Feed Technology Co. Ltd, Yantai 265500, China

## Abstract

A 3 × 2 factorial experiment (protein levels, 42%, 46%, 50%; lipid levels, 9%, 12%) with three replicates was conducted in a circulating water system to investigate the effects of dietary protein and lipid levels on growth, feed utilization, body composition, and serum biochemical parameters of growing rockfish *Sebastes schlegeli* (initial weight, 29.98 ± 0.10 g). After an 8 weeks feeding trial, growth performance in terms of final body weight, percent weight gain, and specific growth rate increased with the increase of dietary protein level when fish fed diets containing a consistent level of dietary lipid. The feed conversion rate and daily feed intake were significantly affected by dietary protein and lipid levels, and decreased as dietary protein level increased from 42% to 46% or dietary lipid level increased from 9% to 12% (*P* < 0.05). Survival rate, viscerosomatic index, and hepatosomatic index were unaffected by dietary protein level (*P* > 0.05), but significantly increased with the increase of dietary lipid level (*P* < 0.05). On the contrary, condition factor was unaffected by dietary lipid level (*P* > 0.05), but significantly increased with dietary protein level increasing up to 46% (*P* < 0.05). The moisture contents of muscle and liver significantly decreased, but the whole-body crude lipid content, the crude protein and lipid contents of muscle increased as dietary protein or lipid level increased (*P* < 0.05). The contents of isoleucine, leucine, histidine, glycine, alanine of muscle, as well as the proportions of C14 : 0, C20 : 1, and C22 : 1n-9 in total fatty acids were higher in fish fed diets containing 12% lipid than those fed 9% lipid (*P* < 0.05), while C18 : 1n-9 and C18 : 2n-6 followed an opposite trend. The contents of phenylalanine, lysine, and tyrosine as well as the proportions of C18 : 0, C18 : 2n-6, C22 : 1n-9, and C22 : 6n-3 in total fatty acids decreased with the increase of dietary protein level (*P* < 0.05). Serum cholesterol and low-density lipoproteins increased significantly with dietary protein or lipid levels increasing, but TG concentration was elevated significantly in fish fed diets containing 12% lipid. Considering the present results in terms of growth and feed utilization, the suitable protein and lipid levels in diet for growing rockfish were 46% and 12%, respectively.

## 1. Introduction

Dietary protein and lipid are two expensive macronutrients in fish aquafeeds affecting fish growth performance and feed cost [1]. Due to the poor carbohydrate utilization by fish, especially by carnivorous fish, the energy needed for growth and metabolism is mainly provided by dietary protein and lipid [2]. Without adequate alternative energy sources (lipid) to meet energy demands in feed, some of the dietary protein consumed have to be degraded to support the energy demands for tissue synthesis and metabolism, resulting in a high protein requirement. Lipid has more than twice as many calories per gram as carbohydrate and protein [3]. Therefore, sufficient lipid sources are supplemented in the feed to meet general energy requirement, allowing fish to direct the maximum level of available dietary protein to growth. This is defined as protein-sparing effect of lipid, which is beneficial to reduce feed cost and nitrogenous waste output in fish farming [4]. In recent years, some research findings have evidenced that diets with appropriate protein and lipid levels are performing well in terms of fish growth and feed utilization, while a sparing effect on protein by increased dietary lipid has also been found in several fish [5–7]. However, most commercial feeds containing relatively high level of protein and lipid are applied in offshore cage farming, resulting in feed waste, and potential environmental pollution. Moreover, dietary protein and lipid levels also affect tissue lipid accumulation, health status, and basal metabolism, and consequently influence fish survival.

Rockfish is an economically important marine carnivorous fish, widely distributed in Japan, Korea, and northeast coast of China [8]. In recent years, its wild population has declined rapidly in some areas because of overfishing [9]. However, rising fish consumption has led to increased focus on production of fish in cages. Rockfish is a suitable species for offshore cage culture and stock enhancement for its high growth rate, disease resistance, and cold tolerance. Because of its economic and ecological importance, efforts have been made to improve the productivity of rockfish, including seed production, nutrition regulation, vaccine development, and so on. The recent research on nutritional regulation mainly focuses on dietary macronutrients requirements, especially protein (54.0%, [10]) and lipid (17.3%, [11]). In addition, an early study reported that the optimum protein and lipid levels for growth and feed utilization of rockfish fry were 50% and 15%, 45% and 19%, pointing to the obvious protein-sparing effect of lipid [12]. Small rockfish with an initial weight of less than 3.0 g were used in the above experiments. In China, however, large-size fries (>30 g) are preferred for offshore cage culture due to the excellent environmental adaptability and high survival. Dietary protein and lipid requirements are influenced by fish size, environment, and feed formulation. Up to now, there were no reports regarding to the proportion optimization of dietary protein and lipid for large-size rockfish fries. Thus, further research is required for the development of rockfish feed with an optimal balance between protein and lipid contents, achieving an efficient use of dietary protein. The aim of this study is to obtain an economically acceptable formula with an optimal proportion of protein and lipid, by investigating the effects of different dietary protein and lipid levels on growth, feed utilization, body composition, and serum biochemical parameters.

## 2. Materials and Methods

### 2.1. Experimental Diets

Six experimental diets were formulated in a 3 × 2 factorial design to include three protein levels (42%, 46%, and 50%) and two lipid levels (9% and 12%), producing P/E ratios in the range of 21.99–27.07 mg protein kJ^−1^ (Table 1). Fishmeal, soybean meal hydrolysate, and soybean protein concentrate were used as the main protein sources and incorporated in a fixed proportion to ensure the same amino acid pattern in all diets. Fish oil was used as the single lipid source for energy. Crystalline methionine was added in all test diets to avoid methionine deficiency. The solid ingredients were ground with a grinder to pass through a 60 mesh sieve. The trace components were mixed by gradually expanding. All ingredients were thoroughly mixed in a feed mixer, and then fish oil and distilled water were added and mixed to homogeneity. The mixtures were then extruded into 3.0 mm pellets with a double-screw extruder machine (G-250, machine factory of South China University of Technology, Guangzhou, China). All pellets were placed in a forced ventilation oven at 60°C and air-dried to approximately 6% moisture. Dried diets were sealed in plastic bags and stored at −20°C until used. The formula and proximate composition of the experimental diets are shown in Table 1. Dietary amino acids compositions and fatty acids proportions in total fatty acids are shown in Table 2 and Table 3, respectively.

### 2.2. The Feeding Trial Management

The feeding trial was conducted in a recirculating aquaculture system in Dongying Experimental Base of Shandong Marine Resource and Environment Research Institute (Yantai, China). Rockfish were purchased from a commercial fish farm (Weihai, China). Prior to the start of the experiment, fish were fed a commercial diet for 2 weeks and acclimated to the experimental conditions. Thereafter, 540 rockfish with similar sizes (initial average weight, 29.98 ± 0.10 g) were randomly assigned to 18 fiber glasstanks (L-100 cm, W-50 cm, H-80 cm) with a density of 30 fish per tank. Each experimental diet was fed randomly to triplicate tanks of fish. All fish were fed two times daily (8 : 00 and 16 : 00) to apparent satiation, and the feed intake was recorded. During the 56 days trial, water temperature was maintained at 17°C ± 1, pH between 7.0 and 7.5, salinity 27.0 ± 1.00, unionized ammonia nitrogen <0.05 mg L^−1^, and dissolved oxygen >5.0 mg/L. The water quality parameters were monitored periodically.

### 2.3. Sample Collections

At the end of the feeding trial, fish in each tank were starved for 24 hr. The total number and final weight (FW) of rockfish in each tank were measured. Fifteen fish were randomly taken from each tank and anesthetized with MS-222 (3-aminobenzoic acid ethyl ester methanesulfonate, 45 mg/L) prior to sampling. Five out of 15 fish were used for body composition analysis. Other 10 fish were individually measured for body weight and length, collected blood with a syringe from the caudal vein, and then were dissected for viscera, liver, and muscle. Blood samples were centrifuged at 4,000 g under 4°C for 10 min (centrifugeCT15RE; Hitachi, Tokyo, Japan). The serum was separated and stored at −80°C until analysis of serum biochemical parameters. All tissue samples were frozen immediately in liquid nitrogen and stored at −80°C for the analysis of proximate composition.

### 2.4. Growth Calculation

The growth parameters and diet utilization were calculated according to the following formulas:

  Weight gain rate (WGR, %) = (final weight - initial weight) (g)/initial weight (g) × 100;
  Specific growth rate (SGR, %/d) = (Ln final weight - Ln initial weight)/days of experiment × 100;
  Feed conversion ratio (FCR) = dry feed intake (g)/weight gain (g);
  Daily feed intake (DFI, %/d) = dry weight of consumed feed (g)/((initial weight + final weight)/2 ×days) × 100;
  Protein efficiency ratio (PER) = weight gain (g)/ingested protein (g);
  Hepatosomatic index (HSI, %) = hepatopancreas weight (g)/whole body weight (g) × 100;
  Viscerosomatic index (VSI, %) = viscera weight (g)/whole body weight (g) × 100;
  Condition factor (CF, g/cm^3^) = whole body weight (g)/body length (cm)^3^;
  Survival rate (SR, %) = final amount of fish/initial amount of fish × 100.

### 2.5. Proximate Composition Analysis

Proximate compositions of diets, muscle, liver, and whole body were analyzed according to the standard methods of Official Analytical Chemists [13]. Moisture content was determined by drying the samples to a constant weight in an oven (105°C). Crude protein (*N* × 6.25) was determined using the Kjeldahl method after an acid digestion. Crude lipid was analyzed by the ether extraction method using the Soxtec System HT. Crude ash was determined using a muffle furnace (Linder/blue M1100, Thermo Fisher Scientific Co., Ltd., China) at 550°C for 6 hr. Total energy was measured with an automatic bomb calorimeter (IKA C6000, Aika Instrument and Equipment Co., Ltd., Guangzhou). Amino acids compositions of muscle and diets were analyzed using HCl [14]. In brief, the hydrolysis (6 mol/L HCl) of the samples was performed in Pyrex microcapillary tubes (Pierce Chemical Company, Rockford, IL, USA) under vacuum and heated at temperatures (110°C) for 22 hr. After hydrolysis, the samples were filtered using Spartan-HPLC 13 mm syringe filters (0.45 *μ*m, 30 mm; Schleicher and Schuell, Dassel, Germany) and analyzed by an automatic amino acid analyzer (Hitachi L-8900, Japan). Fatty acids were analyzed according to the method of Metcalfe et al. [15]. In brief, total lipids were extracted using hexane as a solvent, and then hydrogen chloride methanol solution (acetyl chloride: methyl alcohol = 1 : 10) was added to saponify total lipids and derivatized them into fatty acid methyl esters at 80°C under the catalysis of K_2_CO_3_. These fatty acid methyl esters were analyzed with gas chromatography (GC-2010, Hitachi, Japan) and were identified by comparison of their retention times with known standards (Supelco, Bellefonte, PA, USA).

### 2.6. Activity Analyses of Serum Biochemical Indices

Serum total protein (TP) was determined using Coomassie Brilliant Blue G-250 dye-binding technique of Bradford [16]. Triglycerides (TGs) were analyzed using glycerol dehydrogenase and a water-soluble formazan dye according to the methods of Kawano et al. [17]. Cholesterol (CHO) was analyzed using enzymatic colorimetric method of Robinet et al. [18] by calculating the difference between the total and free cholesterol contents. Albumin (ALB) and high-density lipoprotein (HDL) were analyzed using the commercial kits purchased from Nanjing Jiancheng Bioengineering Institute (Nanjing, Jiangsu, China). Albumin (ALB) colorimetric assay was based on the selective interaction between bromocresol green and albumin forming a chromophore that could be detected at 620 nm. High-density lipoprotein (HDL) measurement used sulfated alpha-cyclodextrin in the presence of Mg^2+^, which formed complexes with apoB-containing lipoproteins, and polyethylene glycol-coupled cholesteryl esterase and cholesterol oxidase. LDL-cholesterol was calculated from measured values of total cholesterol, triglycerides, and HDL-cholesterol according to the relationship: (LDL-chol) = (total chol)-(HDL-chol)-(TG)/5. The activities of aspartate aminotransferase (AST) and alanine transaminase (ALT) were also determined using the commercial kits. One unit of AST is the amount of enzyme that will generate 1.0 mol of glutamate per minute at pH 8.0, 37°C. One unit of ALT is defined as the amount of enzyme that generates 1.0 mol of pyruvate per minute at 37°C.

### 2.7. Statistical Analysis

All data were expressed as means ± standard deviation and subjected to one- and two-way ANOVA analyses to determine whether there were significant differences due to the dietary levels of protein, lipid or the interaction. If significant differences were found (*P* < 0.05), Duncan's multiple range test was used to compare the mean values between individual treatment. All statistical analyses were carried out by using the SPSS program Version 16.0 (SPSS Inc., Chicago, IL, USA) for Windows.

## 3. Results

### 3.1. Growth and Feed Utilization

The growth performance and feed utilization were presented in Table 4. Analysis of two-way ANOVA showed FBW, WGR, and SGR increased with dietary protein level increasing from 42% to 50%, while FCR and DFI followed an opposite trend. Fish fed diets containing 46% and 50% protein had higher FBW, WGR, and SGR and lower FCR and DFI than those fed diets containing 42% protein (*P* < 0.05). However, there were no statistically significant differences in the parameters between 46% and 50% protein dietary treatments (*P* > 0.05). No difference was detected in PER among all treatments (*P* > 0.05). Diets containing 12% lipid significantly reduced FCR and DFI but increased significantly HIS, VSI, and SR compared to diets containing 9% lipid (*P* < 0.05). CF increased with increasing dietary protein level from 42% to 46% (*P* < 0.05). However, a further increase in dietary protein level to 50% did not support the further increase in CF (*P* > 0.05).

### 3.2. Body Composition

The proximate compositions of whole body, dorsal muscle, and liver were presented in Table 5. The crude protein contents of liver and whole body were not affected by dietary protein and lipid levels (*P* > 0.05). The crude lipid content of liver increased with the dietary protein or lipid level increasing while the moisture contents of muscle and liver followed an opposite trend. The whole-body crude lipid content, the crude protein content, and the crude ash content of muscle were not altered as dietary protein increased from 42% to 46% (*P* > 0.05), but significantly increased as dietary protein increased to 50% (*P* < 0.05). The crude ash content of liver significantly decreased but crude protein content of muscle, and crude lipid contents of whole body, muscle, and liver increased in fish fed diets containing 12% lipid as compared to those fed diets with 9% lipid (*P* < 0.05).

### 3.3. Amino Acids Compostions and Fatty Acids Profile of Muscle

The amino acids compositions of muscle were presented in Tables 6 and 7, the contents of isoleucine, leucine, histidine, glycine, and alanine of muscle were higher in fish fed diets containing 12% lipid than those fed diets containing 9% lipid (*P* < 0.05). Fish fed diets containing 50% protein had higher histidine contents but lower phenylalanine and lysine contents compared to those fed diets containing 42% protein (*P* < 0.05). The fatty acids profile of muscle was presented in Table 8. The proportions of C16 : 0, C20 : 4n-6 (arachidonic acid), and C20 : 5n-3 (eicosapentaenoic acid) were not altered regardless of dietary protein or lipid level (*P* > 0.05). The C14 : 0, C20 : 1, and C22 : 1n-9 were increased but C18 : 1n-9 (oleic acid) and C18 : 2n-6 (linoleic acid) were decreased in fish fed diets containing 12% lipid compared to those fed diets containing 9% lipid (*P* < 0.05). C18 : 0 and C22 : 1n-9 decreased significantly with dietary protein level increasing but C18 : 1n-9 followed an opposite trend. Fish fed diets containing 42% protein had lower C16 : 1 proportion but higher C22 : 6n-3 (docosahexaenoic acid (DHA)) proportion than those fed diets containing 46% and 50% protein (*P* < 0.05), and fish fed diets containing 50% protein had lower C18 : 2n-6 than those fed diets containing 42% and 46% protein (*P* < 0.05).

### 3.4. Serum Parameters

As summarized in Table 9, serum AST, ALT, TP, ALB, and HDL were similar among all dietary treatments (*P* > 0.05). The contents of serum CHO and LDL were significantly affected by dietary protein and lipid levels (*P* < 0.05). Serum CHO and LDL levels increased significantly in fish fed diets with 50% protein as compared to that fed diets with 42% and 46% protein (*P* < 0.05) and also increased in fish fed diets containing 12% lipid than those fed diets containing 9% lipid (*P* < 0.05). Serum TG content was not affected by dietary protein levels (*P* > 0.05) but was elevated significantly as dietary lipid increased from 9% to 12% (*P* < 0.05).

## 4. Discussion

After 8 weeks feeding trial, the growth performance of growing rockfish in terms of SGR ranged within 1.31–1.44%/d for 29.98 g and represented a satisfactory level in comparison with previous investigations on rockfish of similar size (0.52–1.07%/d for 43.61 g, [19]; 0.21%/d for 38.0 g, [20]). However, the present growth response of growing rockfish in terms of WGR and SGR suggested dietary protein requirement of rockfish (29.98 g) was about 46%, because no further significant gains in growth performance were observed as dietary protein level increased from 46% to 50%. The predicted value was closed to the protein requirement (44%) of rockfish with initial weight of 21.9 g reported by Lee et al. [21], but much lower than those reported in previous studies for smaller rockfish (48.6%–50% for 7.3 g, 54% for 10 days larvae), indicating that larger rockfish required less dietary protein than did the smaller rockfish. In addition, soy protein hydrolysates have been proved to improve protein availability and promote growth performance of starry flounder *Platichthys stellatus* and turbot *Scophthalmus maximus* in our previous studies [22–24]. In the present study, hydrolyzed soybean meal was incorporated into the experimental diets to improve protein availability and thus might reduce dietary protein requirement.

There is some inconsistency with regard to reporting of dietary lipid requirement of rockfish. Kim et al. [11] reported rockfish larvae (10 days) required 17.3% lipid in microdiet containing 52.4%–52.9% protein to support their development. Aminikhoei et al. [25] reported that 12% lipid in diets containing 52% protein was sufficient for the growth of rockfish (1.7 g). This suggested dietary lipid requirement decreased as rockfish grew. In the present study, the increasing dietary lipid from 9% to 12% did not result in a significant enhancement of growth performance of rockfish (29.98 g). This indicated that 9%–12% of dietary lipid had met the energy requirement of rockfish which was consistent with the review of Lee [26]. On the other hand, the protein-sparing effect is observed by a concomitant decrease in dietary protein and increase in dietary lipid and is more pronounced at the suboptimum level of dietary protein and higher level of lipid [27, 28], which is not the case in the present study as the best growth was recorded in fish fed diet P50L12 while not in fish fed diet P42L12 and P46L12. Therefore, the present findings indicated growing rockfish had limited ability to oxidize lipid and relied more heavily on protein as a primary energy source, suggesting the lack of the protein-sparing action of lipid. Less energy derived from dietary lipid was deposited in the form of protein, but proportionally more was deposited as lipid reserves and weight increase of lipid in fish was not enough to significantly affect rockfish growth. Contrary to the present findings, Lee et al. [21] and Cho et al. [12] reported the increasing 4%–7% lipid could spare about 5% protein in diet for growing rockfish (21.9 g and 3.2 g, respectively), and thus they estimated a high dietary lipid requirement (14%–19%). Considering the difference in lipid sources in these studies, rockfish might utilize fish oil more efficiently than the mixture of fish oil and soybean oil and thus require less lipid to support growth, explaining a low lipid requirement of rockfish in the present study.

In the present study, all tested diets were well-accepted by the fish, with DFI values ranging from 1.67%/d to 1.76%/d, representing a satisfactory palatability compared to that of 0.92%/d–1.06%/d reported by Lee et al. [21]. However, fish DFI decreased at higher protein and lipid levels which agreed with the studies in brown-marbled grouper *Epinephelus fuscoguttatus* [29], silver sillago *Sillago sihama* [30], European grayling *Thymallus thymallus* [31], indicating that feed intake was regulated by the dietary available energy. Generally, the increased diet energy content can lead to lesser diet being consumed by fish to meet its energy requirement. On the contrary, when fish are offered diets with an energy content below the requirement level, they would consume more feed to gain sufficient energy needed for supporting growth and metabolism. The DFI response suggested diets containing 46%–50% protein and 12% lipid provided 18.47–18.96 kJ/g energy, which met the energy needs of fish and significantly reduced diet consumption. It was noted the feed conversion rate decreased with the increasing protein and lipid levels, which is consistent with studies on rockfish [21], Manchurian trout *Brachymystax lenok* [32], brown trout *Salmo trutta fario* [33], black sea bass *Centropristis striata* [34], and European grayling *T. thymallus* [35]. The increased lipid level improved feed utilization and consequently diet P46L12 achieved a similar performance (FCR) to diet P50L12, pointing out an obvious protein-sparing effect of lipid [21]. In addition, the current result showed that PER was unaffected regardless of dietary protein or lipid level. This meant that dietary protein of 42%–50% were exactly deposited in proportion to weight growth and thus no obvious PER response occurred, which was not inconsistent with results reported by Cho et al. [12] and Lee et al. [21]. To sum up, the suitable dietary protein and lipid ranged within 46%–50% and 12%, respectively, with the energy level above 18.47–18.96 kJ/g, to achieve minimum feed consumption and maximum feed utilization.

Allometric growth of tissue is a long-term process of adapting to external stimuli including nutritive stimuli. Morphometrical parameters, such as HSI, VSI, and CF, are often used as indicators to assess the nutritional status of fish [36]. In the present study, high-lipid (12%) diets increased HSI and VSI, which consisted with some findings in red-spotted grouper [37], northern whiting *S. sihama* [38], and haddock *Melanogrammus aeglefinus* [39]. The significantly increased HSI and VSI in fish fed the high-lipid (12%) diets was associated with the increasing lipid accumulation in fish body, as presented in Table 5, which explained by low lipid transport out of liver or limited lipid catabolic activity [40, 41], as concluded in growth response. In addition, hepatic lipid accumulation is considered as a symptom of fatty liver [40]. However, no obvious symptom of liver injury was found in the present study, since activities of serum transaminases were not elevated as dietary lipid increased. Furthermore, appropriate deposition of lipid in liver was beneficial for fish to cope with unfavorable stimuli [42, 43]. The present results corroborated these findings and indicated survival rate significantly increased as dietary lipid increasing from 9% to 12%, suggesting that dietary lipid sources may affect survivability of rockfish as demonstrated in other fish [42].

An increased in lipid contents coupled with a decrease in moisture content with increasing dietary lipid at each protein level in whole body, muscle, and liver, which were consistent with the results reported in African catfish *Clarias gariepinus* [44], surubim *Pseudoplatystoma coruscans* [45], bagrid catfish *Pseudobagrus fulvidraco* [46], and *Nibea diacanthus* [47]. High inclusion level of dietary protein (50%) significantly promoted lipid deposition in liver and whole body but did not affect protein deposition. This was in line with the findings reported on mangrove red snapper *Lutjanus argentimaculatus* [48], red swamp crayfish *Procambarus clarkia* [49], and topmouth culter *Culter alburnus* [50], suggesting that excess protein may be stored as energy or convert into lipid. In muscle, the increased protein deposition was observed in fish fed diet P46L12, similar to those fed P50L12 and P50L9 but higher than those fed other low-lipid (9%) diets. This indicated that high dietary lipid promoted protein deposition and exhibited an obvious protein-sparing effect.

In the present study, the increment of dietary lipid reduced the proportion of oleic acid and linoleic acid in total fatty acids of muscle, which was in line with that reported in Atlantic cod *Gadus morhua* [51], white seabass *Atractoscion nobilis* [52], Japanese seabass *Lateolabrax japonicus* [53], and orange-spotted grouper [54]. However, the increased dietary protein increased the proportion of oleic acid in muscle lipid but reduced the proportions of linoleic acid and DHA. Fish cannot only obtain oleic acid from diet but also endogenously synthesize oleic acid by converting from stearic acid. The increased dietary lipid might suppress oleic acid synthesis or promote oleic acid oxidation, meanwhile the increased dietary protein possibly reduced the oxidation of oleic acid to supply energy and then resulted in more oleic acid deposition in muscle. In addition, rockfish are unable to synthesize linolenic acid and DHA from precursors since they lack specific elongase and desaturases [26, 55]. Therefore, the present study suggested increased dietary lipid and protein suppressed deposition of linoleic acid and DHA in rockfish muscle. Some opposite results were found in studies on Atlantic cod *Gadus morhua* [51], loach *Misgurnus anguillicaudatus* [19], far eastern catfish *Silurus asotus* [56] and turbot *S. maximus* [57]. Further research is needed to explore the different deposition mechanism of linoleic acid and DHA across fish species.

Muscle amino acid deposition may be influenced by nutrients intake, especially protein content [58]. Excess dietary protein lead to catabolism of amino acids into energy [59]. Fish are able to selectively retain or catabolize specific amino acids according to the dietary protein to energy ratio [60]. In the present study, growing rockfish selectively retained histidine but catabolized phenylalanine, lysine, and tyrosine when they received high-protein diets, which was in accord with the findings recorded in giant trevally *Caranx ignobilis* [61], *N. diacanthus* [47], and chu's croaker *Nibea coibor* [62]. However, growing rockfish selectively retained histidine, leucine, isoleucine, glycine, and alanine when they received high-lipid diets. These different deposition responses of amino acids probably pointed out the specific amino acids requirement for muscle metabolism when rockfish were subjected to different nutritional stimuli. For example, histidine, isoleucine, and leucine participate in lipoprotein assembling [63–65], lipid metabolism-related genes regulation [66], and antilipid peroxidation [67–69]. It is assumed that selective retention of these amino acids by rockfish is necessary for the enhanced lipid metabolism as reflected in serum TG, CHO, and LDL. Therefore, the roles of these amino acids need to be further elucidated.

Blood biochemical parameters and enzyme activities are used as key means of surveying the fish health and nutritional status [54]. Lu et al. [70] reported that lipid accumulation elevated serum AST and ALT activities of blunt snout bream *Megalobrama amblycephala* fed high-lipid diets, which was associated with liver impairment. In the present study, no obvious difference in the activities of AST and ALT among all treatments suggested that liver impairment did not seem to occur in all treatments. The levels of serum TP and albumin are usually correlated to hepatic protein synthesis of fish, excessive, and inadequate dietary protein intake reduces their concentrations [71, 72]. In present study, dietary protein as low as 42% did not suppress the protein synthesis ability of liver. In terms of serum lipid metabolism, serum CHO and LDL levels both increased as dietary protein and lipid increased; however, serum TG level was elevated by dietary lipid, which agreed with the findings reported in grass carp *Ctenopharyngodon idella* [73], grouper *Epinephelus coioides* [74], and red-spotted grouper [37]. LDL is the main transporter of cholesterol to the peripheral tissues, whereas excess tissue cholesterol is returned to the liver by reverse cholesterol transport mediated by HDL [75]. Therefore, rockfish could well-regulate lipid homeostasis by enhancing lipid transportation for deposition and oxidization in liver or peripheral tissues, to relieve stress caused by high protein dietary or lipid.

## 5. Conclusion

In conclusion, diets containing 46%–50% protein at each lipid level provided the satisfactory growth for this species with an obvious protein-sparing effect of lipid on feed utilization. Therefore, the recommended dietary protein and lipid level are 46% and 12%, respectively, to achieve a compromise between growth and feed utilization. Further experiments are required in this area to investigate the effect of long-term feeding the recommended dietary protein and lipid level on fish health, when considering more lipids accumulated in fish fed high-lipid diet.

## Figures and Tables

**Table 1:** Formulation and proximate composition of the experimental diets (% dry basis).

Ingredients	Experimental diets					
	P50L12	P50L9	P46L12	P46L9	P42L12	P42L9
Fish meal^1^	36	36	33	33	30	30
Hydrolyzed soybean meal^2^	12	12	11	11	10	10
Soybean protein concentrate^3^	27	27	24.75	24.75	22.5	22.5
Microcrystalline cellulose^4^	3.69	6.69	9.75	12.75	15.81	18.81
Fish oil^5^	8.14	5.14	8.33	5.33	8.52	5.52
*α*-starch^6^	9	9	9	9	9	9
Vitaminpremix^7^	1	1	1	1	1	1
Mineral premix^8^	1	1	1	1	1	1
Antioxidant^9^	0.05	0.05	0.05	0.05	0.05	0.05
Choline chloride^10^	0.5	0.5	0.5	0.5	0.5	0.5
Betaine^11^	0.5	0.5	0.5	0.5	0.5	0.5
Soybean lecithin^l2^	1	1	1	1	1	1
Methionine^13^	0.12	0.12	0.12	0.12	0.12	0.12
Total	100	100	100	100	100	100
Proximate composition						
Crude protein	50.65	50.38	46.34	46.59	42.70	42.61
Crude lipid	11.85	9.26	11.93	8.61	11.48	8.86
Crude ash	13.56	13.52	12.46	12.51	11.55	11.59
Digestible energy (kJ/g)	18.96	18.05	18.47	17.52	17.80	17.12
P/E (mg kJ^−1^)	26.28	27.07	24.02	25.06	21.99	22.80

^1^Crude protein, 62.53%; crude lipid, 8.5%; Tongri Food Co., Ltd., Tsingdao, China. ^2^Hydrolyzed soybean meal was obtained using soybean meal (SBM) as a substrate according to the method of Song et al. [22], crude protein, 49.15%, crude lipid, 1.16%; protein solubility, 52.26% (molecular mass < 1,000 Da, 25.05%; 3,000–5,000 Da, 16.15%; >5,000 Da, 11.06%). ^3^Crude protein, 63.47%; crude lipid, 0.8%; Shandong Changrun Biology Co., Ltd., Linyi, China. ^4^Carbohydrate, 98%–99%; Linghu Xinwang Chemical Co., Ltd., Huzhou, China. ^5^Rongcheng Litai Fishmeal Co., Ltd., Rongcheng, China. ^6^Shandong Baisheng Biotechnology Co., Ltd., Yanzhou, China. ^7^Vitamin premix (mg/kg or IU/kg diet): vitamin A 7,500.00 IU, vitamin D 1,500.00 IU, vitamin E 60.00 mg, vitamin K3 18.00 mg, vitamin B1 12.00 mg; vitamin B2 12.00 mg, vitamin B12 0.10 mg, pantothenate acid 48.00 mg, niacin 90.00 mg, folic acid 3.70 mg, D-biotin 0.20 mg, pyridoxine 60.00 mg, and vitamin C 310.00 mg. ^8^Mineral premix (mg/kg diet): Zn 35.00 mg, Mn 21.00 mg, Cu 8.30 mg, Fe 23.00 mg, Co 1.20 mg, I 1.00 mg, and Se 0.30 mg. ^9^Ethoxyquin; Weifang Jiayijia Bio-tech Co., Ltd., Weifang, China. ^10^Henan Jin Yuan Biological Technology Co., Ltd., Zhengzhou, China. ^11^Shandong Longxing Biological Engineering Co., Ltd., Jinan, China. ^12^Shandong Longxing Biological Engineering Co., Ltd., Jinan, China. ^13^Jiangxi Baiying Biological Technology Co., Ltd., Nanchang, China.

**Table 2:** Amino acids compositions of the experimental diets (% dry basis).

Ingredients	Experimental diets					
	P50L12	P50L9	P46L12	P46L9	P42L12	P42L9
Threonine	1.95	2.05	1.84	1.81	1.69	1.61
Valine	2.48	2.49	2.26	2.21	2.01	2.05
Methionine	1.54	1.52	1.42	1.47	1.31	1.37
Isoleucine	2.25	2.24	2.06	2.14	1.85	1.88
Leucine	3.61	3.69	3.32	3.32	3.09	3.05
Phenylalanine	2.24	2.27	2.01	2.01	1.79	1.81
Lysine	3.25	3.19	2.94	2.91	2.72	2.65
Histidine	1.13	1.18	1.01	1.01	0.99	0.92
Arginine	3.38	3.45	3.18	3.19	2.87	2.81
Essential amino acids	21.26	21.58	19.54	19.57	17.82	17.65
Proline	1.74	1.73	1.59	1.64	1.45	1.42
Tyrosine	1.62	1.65	1.49	1.42	1.35	1.41
Serine	1.84	1.79	1.69	1.65	1.53	1.49
Glutamic acid	9.74	9.68	8.92	8.95	8.11	8.34
Glycine	2.86	2.89	2.62	2.67	2.38	2.32
Alanine	2.99	3.05	2.74	2.69	2.49	2.47
Cysteine	0.64	0.62	0.59	0.58	0.54	0.52
Aspartic acid	5.09	4.95	4.67	4.63	4.24	4.18
Nonessential amino acids	26.52	26.36	24.31	24.23	22.09	22.15
Total amino acids	47.78	47.94	43.85	43.8	39.91	39.8

**Table 3:** Fatty acids proportions of the experimental diets (% total fatty acids).

Ingredients	Experimental diets					
	P50L12	P50L9	P46L12	P46L9	P42L12	P42L9
C14 : 0	7.43	7.49	7.48	7.38	7.34	7.42
C16 : 0	17.44	17.48	17.31	17.37	17.34	17.46
C18 : 0	4.08	4.02	3.98	3.91	4.05	3.95
Total saturated fatty acids	34.59	34.55	34.62	34.68	34.67	34.52
C16 : 1	10.42	10.52	10.58	10.54	10.48	10.57
C18 : 1n-9c	11.62	11.68	11.57	11.51	11.59	11.68
C20 : 1	1.53	1.55	1.67	1.65	1.69	1.51
C22 : 1n-9	1.28	1.19	1.25	1.28	1.17	1.11
Total monounsaturated fatty acids	24.82	24.89	24.99	24.97	24.85	24.80
C18 : 2n-6c	1.27	1.22	1.15	1.18	1.22	1.24
C20 : 4n-6	0.12	0.11	0.11	0.10	0.09	0.10
C20 : 5n-3	16.88	16.97	16.99	17.12	17.01	17.08
C22 : 6n-3	8.85	8.84	8.75	8.76	8.82	8.75
Total polyunsaturated fatty acids	27	27.03	26.89	27.06	27.05	27.07

**Table 4:** Effects of different dietary protein and lipid levels on growth performance and feed utilization of growing rockfish.

Groups	FBW^1^(g)	WGR^2^(%)	SGR^3^(%/d)	FCR^4^	DFI^5^(%/d)	PER^6^	SR^7^(%)	HSI^8^(%)	VSI^9^(%)	CF^10^(g/cm^3^)
P50L12	67.12 ± 1.98^b^	123.53 ± 6.04^b^	1.43 ± 0.04^b^	1.23 ± 0.06^a^	1.67 ± 0.03^a^	1.70 ± 0.08	100.0 ± 0.0	2.62 ± 0.18^b^	9.37 ± 0.63^b^	3.17 ± 0.22^b^
P50L9	67.08 ± 1.85^b^	124.42 ± 7.11^b^	1.44 ± 0.05^b^	1.28 ± 0.02^ab^	1.70 ± 0.01^ab^	1.62 ± 0.02	97.33 ± 2.31	2.52 ± 0.19^ab^	8.63 ± 0.74^a^	3.35 ± 0.24^c^
P46L12	65.41 ± 2.01^ab^	117.85 ± 6.87^ab^	1.39 ± 0.06^ab^	1.30 ± 0.12^ab^	1.68 ± 0.06^ab^	1.73 ± 0.16	98.67 ± 2.31	2.68 ± 0.19^b^	9.05 ± 0.72^b^	3.40 ± 0.28^c^
P46L9	65.58 ± 1.09^ab^	118.88 ± 3.79^ab^	1.40 ± 0.03^ab^	1.35 ± 0.03^bc^	1.74 ± 0.01^bc^	1.64 ± 0.04	97.33 ± 2.31	2.34 ± 0.16^a^	8.37 ± 0.57^a^	3.17 ± 0.29^b^
P42L12	63.08 ± 2.26^a^	110.85 ± 8.37^a^	1.33 ± 0.07^a^	1.36 ± 0.07^bc^	1.72 ± 0.02^abc^	1.77 ± 0.10	100.0 ± 0.0	2.53 ± 0.20^ab^	9.29 ± 0.69^b^	3.02 ± 0.29^ab^
P42L9	62.62 ± 1.42^a^	108.56 ± 4.29^a^	1.31 ± 0.04^a^	1.45 ± 0.02^c^	1.76 ± 0.02^c^	1.67 ± 0.02	97.33 ± 2.31	2.28 ± 0.18^a^	8.51 ± 0.61^a^	3.02 ± 0.31^a^
Main effects										
Protein level										
50	67.10^B^	123.97^B^	1.44^B^	1.25^A^	1.68^A^	1.66	98.67	2.60	8.99	3.26^B^
46	65.49^B^	118.36^B^	1.39^B^	1.32^A^	1.71^AB^	1.69	98.00	2.53	8.72	3.28^B^
42	62.85^A^	109.70^A^	1.32^A^	1.40^B^	1.74^B^	1.72	98.67	2.42	8.92	3.02^A^
Lipid level										
12	65.20	117.40	1.39	1.29^A^	1.69^A^	1.73	99.56^B^	2.60^B^	9.23^B^	3.19
9	65.09	117.28	1.38	1.36^B^	1.73^B^	1.65	97.33^A^	2.41^A^	8.50^A^	3.17
Two-way ANOVA (*P*-value)										
Protein	0.005	0.007	0.006	0.005	0.025	0.491	0.783	0.120	0.172	0.000
Lipid	0.901	0.969	0.968	0.048	0.019	0.054	0.028	0.014	0.000	0.725
Interaction	0.955	0.875	0.875	0.813	0.795	0.948	0.783	0.094	0.966	0.002

*Notes*: Values in the same column with different superscript letters show significant difference (*P* < 0.05). ^1^Final body weight (FBW, g); ^2^weight gain rate (WGR,%); ^3^specific growth rate (SGR, %/d); ^4^feed conversion ratio (FCR); ^5^daily feed intake (DFI, %/d); ^6^protein efficiency ratio (PER); ^7^survival rate (SR, %); ^8^hepatosomatic index (HSI,%); ^9^viscerosomatic index (VSI, %); and ^10^condition factor (CF, g/cm^3^).

**Table 5:** Effects of different dietary protein and lipid levels on the body composition of growing rockfish.

Groups	Whole body(%)				Muscle(%)				Liver(%)			
	Moisture	Crude protein	Crude lipid	Crude ash	Moisture	Crude protein	Crude lipid	Crude ash	Moisture	Crude protein	Crude lipid	Crude ash
P50L12	68.75 ± 0.95^a^	16.64 ± 0.21	10.28 ± 0.95^b^	4.11 ± 0.13^a^	76.59 ± 0.12^a^	20.44 ± 0.17^c^	2.51 ± 0.13^b^	1.32 ± 0.05^b^	66.19 ± 0.82^a^	10.86 ± 0.52	15.52 ± 0.73^d^	1.02 ± 0.06^a^
P50L9	70.05 ± 0.30^ab^	16.93 ± 0.35	8.59 ± 0.23^a^	4.23 ± 0.13^ab^	77.24 ± 0.45^ab^	19.55 ± 0.27^b^	2.50 ± 0.22^b^	1.28 ± 0.08^b^	67.42 ± 0.92^ab^	10.81 ± 0.46	12.25 ± 0.54^c^	1.11 ± 0.06^b^
P46L12	69.77 ± 0.61^ab^	16.78 ± 0.37	8.85 ± 0.60^a^	4.32 ± 0.12^b^	77.13 ± 0.65^ab^	19.30 ± 0.20^b^	2.75 ± 0.63^b^	1.17 ± 0.09^a^	67.95 ± 0.65^b^	10.59 ± 0.44	15.45 ± 0.63^d^	1.02 ± 0.05^a^
P46L9	71.38 ± 2.32^b^	16.31 ± 0.89	8.56 ± 0.70^a^	4.21 ± 0.23^ab^	78.93 ± 0.45^c^	18.47 ± 0.35^a^	2.08 ± 0.18^a^	1.15 ± 0.09^a^	69.71 ± 0.82^c^	10.57 ± 0.51	9.52 ± 0.40^b^	1.16 ± 0.07^b^
P42L12	69.53 ± 0.61^ab^	16.62 ± 0.28	9.19 ± 0.66^a^	4.34 ± 0.14^b^	78.13 ± 0.71^bc^	18.83 ± 0.39^a^	2.57 ± 0.23^b^	1.11 ± 0.02^a^	69.94 ± 1.50^c^	11.09 ± 0.37	14.99 ± 0.65^d^	1.10 ± 0.08^ab^
P42L9	69.95 ± 0.47^ab^	16.83 ± 0.49	8.72 ± 0.45^a^	4.26 ± 0.17^ab^	78.63 ± 0.82^c^	18.83 ± 0.25^a^	1.98 ± 0.17^a^	1.15 ± 0.06^a^	70.04 ± 0.46^c^	10.52 ± 0.62	8.30 ± 0.59^a^	1.25 ± 0.05^c^
Main effects												
Protein level												
50	69.40	16.77	9.44^B^	4.17	76.91 ^A^	20.04^B^	2.51	1.30^B^	66.80^A^	10.84	13.88^C^	1.14
46	70.58	16.55	8.74^A^	4.27	78.03^B^	18.88^A^	2.41	1.16 ^A^	68.83^B^	10.58	12.48^B^	1.09
42	69.74	16.73	8.96^A^	4.30	78.38^B^	18.83^A^	2.27	1.13 ^A^	69.99^C^	10.81	11.65^A^	1.17
Lipid level												
12	69.35	16.68	9.44^B^	4.26	77.28^A^	19.52 ^B^	2.61^B^	1.19	68.03^A^	10.85	15.32^B^	1.04^A^
9	70.46	16.68	8.63^A^	4.24	78.27^B^	18.93^A^	2.19^A^	1.19	69.05^B^	10.63	10.02^A^; 02±	1.23^B^
Two-way ANOVA (*P*-value)												
Protein	0.209	0.481	0.007	0.117	0.002	0.000	0.109	0.000	0.000	0.383	0.000	0.480
Lipid	0.055	0.942	0.000	0.679	0.004	0.000	0.000	0.837	0.035	0.202	0.000	0.002
Interaction	0.638	0.142	0.004	0.160	0.144	0.004	0.008	0.334	0.316	0.309	0.000	0.779

*Note*: Values in the same column with different superscript letters show significant difference (*P* < 0.05).

**Table 6:** Effects of different dietary protein and lipid levels on the essential amino acids compositions of muscle of growing rockfish (% dry material).

Groups	Threonine	Valine	Methionine	Isoleucine	Leucine	Phenylalanine	Lysine	Histidine	Arginine	Total
P50L12	4.21 ± 0.07^a^	3.82 ± 0.17^a^	1.33 ± 0.11	3.75 ± 0.19^b^	7.33 ± 0.14^b^	3.60 ± 0.07^a^	7.51 ± 0.14^a^	2.28 ± 0.04^b^	5.07 ± 0.08^a^	38.90 ± 1.89^bc^
P50L9	4.08 ± 0.14^a^	3.78 ± 0.18^a^	1.22 ± 0.11	3.38 ± 0.21^a^	6.81 ± 0.23^a^	3.96 ± 0.11^bc^	7.67 ± 0.27^a^	1.57 ± 0.09^a^	4.99 ± 0.19^a^	37.45 ± 1.35^ab^
P46L12	4.01 ± 0.10^a^	3.69 ± 0.16^a^	1.24 ± 0.05	3.28 ± 0.20^a^	6.67 ± 0.16^a^	3.86 ± 0.08^b^	7.53 ± 0.24^a^	1.61 ± 0.43^a^	4.93 ± 0.13^a^	36.81 ± 1.25^ab^
P46L9	4.46 ± 0.32^b^	3.97 ± 0.29^ab^	1.30 ± 0.12	3.55 ± 0.32^ab^	7.33 ± 0.50^b^	4.14 ± 0.26^cd^	8.23 ± 0.56^b^	1.83 ± 0.51^a^	5.48 ± 0.45^b^	40.28 ± 2.53^cd^
P42L12	4.52 ± 0.23^b^	4.12 ± 0.27^b^	1.23 ± 0.09	3.74 ± 0.25^b^	7.50 ± 0.36^b^	4.32 ± 0.27^d^	8.51 ± 0.41^b^	1.64 ± 0.21^a^	5.56 ± 0.25^b^	41.13 ± 2.05^d^
P42L9	4.00 ± 0.14^a^	3.70 ± 0.17^a^	1.21 ± 0.12	3.31 ± 0.18^a^	6.61 ± 0.21^a^	3.91 ± 0.12^b^	7.50 ± 0.25^a^	1.45 ± 0.09^a^	4.86 ± 0.19^a^	36.57 ± 1.25^a^
Main effects										
Protein level										
50	4.14	3.80	1.28	3.56	7.07	3.78^A^	7.59^A^	1.92^B^	5.03	38.17
46	4.23	3.83	1.27	3.42	7.00	4.00^B^	7.88^B^	1.72^AB^	5.20	38.55
42	4.26	3.91	1.22	3.52	7.06	4.11^B^	8.01^B^	1.55^A^	5.21	38.85
Lipid level										
12	4.25	3.88	1.27	3.59^B^	7.17^B^	3.93	7.85	1.84^B^	5.19	38.95
9	4.18	3.82	1.25	3.41^A^	6.92^A^	4.00	7.80	1.61^A^	5.11	38.10
Two-way ANOVA (*P*-value)										
Protein	0.288	0.423	0.368	0.297	0.829	0.000	0.016	0.013	0.148	0.656
Lipid	0.286	0.414	0.566	0.029	0.017	0.188	0.689	0.027	0.345	0.165
Interaction	0.000	0.002	0.135	0.001	0.000	0.000	0.000	0.002	0.000	0.000

*Note*: Values in the same column with different superscript letters show significant difference (*P* < 0.05).

**Table 7:** Effects of different dietary protein and lipid levels on the nonessential amino acids compositions of muscle of growing rockfish (% dry material).

Groups	Proline	Tyrosine	Serine	Glutamic acid	Glycine	Alanine	Cysteine	Aspartic acid	Total
P50L12	2.80 ± 0.08^b^	3.21 ± 0.16^a^	4.02 ± 0.08^a^	13.18 ± 0.31^a^	4.64 ± 0.16^b^	6.04 ± 0.11^b^	1.84 ± 0.06	9.41 ± 0.12^a^	45.13 ± 0.80^b^
P50L9	2.59 ± 0.07^a^	3.37 ± 0.06^ab^	3.85 ± 0.13^a^	12.71 ± 0.46^a^	4.16 ± 0.17^a^	5.53 ± 0.19^a^	1.89 ± 0.08	9.21 ± 0.30^a^	43.31 ± 1.32^ab^
P46L12	2.57 ± 0.08^a^	3.24 ± 0.07^a^	3.80 ± 0.09^a^	12.50 ± 0.29^a^	4.16 ± 0.12^a^	5.46 ± 0.14^a^	1.83 ± 0.12	9.07 ± 0.23^a^	42.64 ± 0.80^a^
P46L9	2.85 ± 0.25^b^	3.51 ± 0.13^bc^	4.26 ± 0.29^b^	13.96 ± 0.98^b^	4.64 ± 0.31^b^	6.05 ± 0.39^b^	1.95 ± 0.12	10.09 ± 0.68^b^	47.32 ± 3.03^c^
P42L12	2.84 ± 0.15^b^	3.65 ± 0.17^c^	4.32 ± 0.24^b^	14.35 ± 0.73^b^	4.68 ± 0.19^b^	6.16 ± 0.31^b^	1.93 ± 0.13	10.29 ± 0.53^b^	48.22 ± 2.36^c^
P42L9	2.49 ± 0.08^a^	3.29 ± 0.16^a^	3.81 ± 0.14^a^	12.70 ± 0.33^a^	4.04 ± 0.16^a^	5.38 ± 0.17^a^	1.82 ± 0.06	9.03 ± 0.28^a^	42.56 ± 1.28^a^
Main effects									
Protein level									
50	2.69	3.29^A^	3.93	12.95	4.40	5.78	1.86	9.31	44.22
46	2.71	3.38^AB^	4.03	13.23	4.40	5.76	1.89	9.59	44.98
42	2.66	3.47^B^	4.06	13.53	4.36	5.77	1.87	9.66	45.39
Lipid level									
12	2.73	3.37	4.04	13.35	4.50^B^	5.89^B^	1.86	9.59	45.33
9	2.64	3.39	3.97	13.12	4.28^A^	5.66^A^	1.88	9.45	44.40
Two-way ANOVA (*P*-value)									
Protein	0.709	0.008	0.201	0.065	0.849	0.965	0.806	0.100	0.284
Lipid	0.051	0.551	0.241	0.256	0.002	0.007	0.605	0.296	0.130
Interaction	0.000	0.000	0.000	0.000	0.000	0.000	0.029	0.000	0.000

*Note*: Values in the same column with different superscript letters show significant difference (*P* < 0.05).

**Table 8:** Effects of different dietary protein and lipid levels on the major fatty acids of growing rockfish (% total fatty acids).

Groups	C14 : 0	C16 : 0	C16 : 1	C18 : 0	C18 : 1n-9c	C18 : 2n-6c	C20 : 1	C20 : 4n-6	C20 : 5n-3	C22 : 1n-9	C22 : 6n-3
P50L12	3.39 ± 0.16^a^	17.91 ± 0.51	5.64 ± 0.32^bc^	3.98 ± 0.14^a^	17.84 ± 0.67^b^	5.37 ± 0.11^a^	2.47 ± 0.10^a^	0.99 ± 0.05	7.49 ± 0.16	2.79 ± 0.34^b^	13.50 ± 0.90^bc^
P50L9	3.40 ± 0.03^a^	18.17 ± 0.14	6.01 ± 0.05^d^	3.94 ± 0.05^a^	19.39 ± 0.23^c^	6.16 ± 0.09^c^	2.29 ± 0.04^a^	0.94 ± 0.01	7.29 ± 0.08	2.55 ± 0.24^ab^	12.05 ± 0.08^a^
P46L12	3.73 ± 0.21^b^	18.15 ± 0.26	5.90 ± 0.30^cd^	4.00 ± 0.19^a^	17.75 ± 0.51^b^	5.59 ± 0.08^b^	2.79 ± 0.47^b^	0.94 ± 0.05	7.39 ± 0.15; 1	3.16 ± 0.34^c^	12.67 ± 1.11^ab^
P46L9	3.32 ± 0.07^a^	18.25 ± 0.20	5.59 ± 0.21^b^	4.28 ± 0.06^b^	18.25 ± 0.33^b^	6.17 ± 0.13^c^	2.36 ± 0.05^a^	0.99 ± 0.06	7.23 ± 0.21	2.56 ± 0.12^ab^	12.70 ± 0.60^ab^
P42L12	3.68 ± 0.17^b^	17.95 ± 0.49	5.29 ± 0.32^a^	4.36 ± 0.15^b^	16.73 ± 0.54^a^	5.60 ± 0.08^b^	2.89 ± 0.37^b^	0.98 ± 0.11	7.40 ± 0.37	3.41 ± 0.15^c^	13.42 ± 1.19^bc^
P42L9	3.26 ± 0.05^a^	18.07 ± 0.34	5.37 ± 0.08^ab^	4.24 ± 0.03^b^	17.82 ± 0.24^b^	6.17 ± 0.06^c^	2.36 ± 0.03^a^	1.02 ± 0.03	7.36 ± 0.09	2.48 ± 0.04^a^	13.74 ± 0.25^c^
Main effects											
Protein level											
50	3.40	18.04	5.82^B^	3.96^A^	18.62^C^	5.76^A^	2.38	0.96	7.39	2.67^A^	12.78^A^
46	3.53	18.20	5.75^B^	4.14^B^	18.00^B^	5.88^B^	2.57	0.96	7.31	2.86^AB^	12.68^A^
42	3.47	17.97	5.33^A^	4.30^C^	17.27^A^	5.88^B^	2.62	1.00	7.38	2.94^C^	13.58^B^
Lipid level											
12	3.60^B^	18.00	5.61	4.11	17.44^A^	5.52^A^	2.72^B^	0.97	7.43	3.12^B^	13.20
9	3.33^A^	18.13	5.66	4.15	18.49^B^	6.17^B^	2.34^A^	0.98	7.30	2.53^A^	12.83
Two-way ANOVA (*P*-value)											
Protein	0.087	0.244	0.000	0.000	0.000	0.005	0.061	0.267	0.606	0.025	0.020
Lipid	0.000	0.257	0.561	0.284	0.000	0.000	0.000	0.456	0.067	0.000	0.180
Interaction	0.001	0.716	0.006	0.001	0.027	0.015	0.226	0.131	0.614	0.005	0.025

*Note*: Values in the same column with different superscript letters show significant difference (*P* < 0.05).

**Table 9:** Effects of different dietary protein and lipid levels on serum biochemical indexes of growing rockfish.

Groups	TP^1^(g/L)	ALB^2^(g/L)	TG^3^(mmol/L)	CHO^4^(mmol/L)	HDL^5^(mmol/L)	LDL^6^(mmol/L)	ALT^7^(U/L)	AST^8^(U/L)
P50L12	34.64 ± 2.63	24.33 ± 1.83	2.60 ± 0.17^c^	5.06 ± 0.14^e^	3.60 ± 0.19	0.67 ± 0.02^e^	29.69 ± 2.29	35.80 ± 1.78
P50L9	36.62 ± 1.47	24.67 ± 1.36	2.39 ± 0.17^ab^	4.78 ± 0.21^d^	3.43 ± 0.19	0.61 ± 0.03^d^	28.99 ± 2.41	34.60 ± 2.94
P46L12	34.15 ± 2.50	24.24 ± 1.83	2.48 ± 0.18^bc^	4.94 ± 0.13^e^	3.48 ± 0.14	0.65 ± 0.02^e^	29.69 ± 2.12	34.01 ± 2.78
P46L9	35.64 ± 2.19	25.58 ± 1.79	2.26 ± 0.17^a^	4.36 ± 0.14^b^	3.50 ± 0.15	0.54 ± 0.01^b^	29.64 ± 2.33	33.75 ± 2.74
P42L12	35.68 ± 2.34	25.23 ± 1.74	2.50 ± 0.19^bc^	4.58 ± 0.16^c^	3.57 ± 0.13	0.58 ± 0.01^c^	28.01 ± 1.63	35.72 ± 2.99
P42L9	34.79 ± 2.60	24.04 ± 1.64; 04	2.26 ± 0.16^a^	3.89 ± 0.14^a^	3.45 ± 0.18	0.47 ± 0.02^a^	28.44 ± 2.05	33.26 ± 1.49
Main effects								
Protein level								
50	35.63	24.50	2.50	4.92^B^	3.52	0.64^C^	29.34	35.20
46	34.90	24.91	2.37	4.48^A^	3.49	0.60^B^	29.66	33.88
42	35.24	24.63	2.38	4.42^A^	3.51	0.52^A^	28.22	34.49
Lipid level								
12	34.82	24.60	2.53^B^	4.84^B^	3.55	0.63^B^	29.13	35.18
9	35.69	24.76	2.30^A^	4.38^A^	3.46	0.54^A^	29.02	33.87
Two-way ANOVA (*P*-value)								
Protein	0.645	0.272	0.064	0.000	0.868	0.000	0.120	0.299
Lipid	0.180	0.117	0.000	0.000	0.066	0.000	0.858	0.064
Interaction	0.153	2.505	0.978	0.060	0.225	0.000	0.733	0.431

*Notes*: Values in the same column with different superscript letters show significant difference (*P* < 0.05). ^1^Total protein (TP, g/L); ^2^albumin (ALB, g/L); ^3^triglyceride (TG, mmol/L); ^4^cholesterol (CHO, mmol/L); ^5^high-density lipoprotein (HDL, mmol/L); ^6^low-density lipoprotein (LDL, mmol/L); ^7^alanine transaminase (ALT, U/L); and ^8^aspartate aminotransferase (AST, U/L).

## Data Availability

The authors confirm that the data supporting the findings of this study are available within its supplementary material.

## Supplementary Materials

Supplementary Materials All experimental data were presented in the supplementary file, including growth performance and diet utilization, hepatosomatic index, viscerosomatic index, condition factor, whole body proximate compositions, muscle proximate compositions, liver proximate compositions, essential amino acid composition of muscle, nonessential amino acid composition of muscle, major fatty acids of muscle, and serum biochemical indexes of rockfish *Sebastes schlegeli*. Table S1: growth performance and feed utilization. Table S2: hepatosomatic index (%). Table S3: viscerosomatic index (%). Table S4: condition factor (CF, g/cm^3^). Table S5: whole body proximate compositions (%). Table S6: muscle proximate compositions (%). Table S7: liver proximate compositions (%). Table S8: essential amino acid composition of muscle (%). Table S9: nonessential amino acid composition of muscle (%). Table S10: major fatty acids of muscle. Table S11: serum biochemical indexes.
