# Supplementary material for: Effects of Dietary Protein and Lipid Levels in Practical Formulation on Growth, Feed Utilization, Body Composition, and Serum Biochemical Parameters of Growing Rockfish Sebastes schlegeli
Source: Aquac Nutr. 2023 Aug 8;2023:9970252. doi: 10.1155/2023/9970252 (PMC10427231; doi:10.1155/2023/9970252)
Supplement: Supplementary Materials — All experimental data were presented in the supplementary file, including growth performance and diet utilization, hepatosomatic index, viscerosomatic index, condition factor, whole body proximate compositions, muscle proximate compositions, liver proximate compositions, essential amino acid composition of muscle, nonessential amino acid composition of muscle, major fatty acids of muscle, and serum biochemical indexes of rockfish Sebastes schlegeli. Table S1: growth performance and feed utilization. Table S2: hepatosomatic index (%). Table S3: viscerosomatic index (%). Table S4: condition factor (CF, g/cm3). Table S5: whole body proximate compositions (%). Table S6: muscle proximate compositions (%). Table S7: liver proximate compositions (%). Table S8: essential amino acid composition of muscle (%). Table S9: nonessential amino acid composition of muscle (%). Table S10: major fatty acids of muscle. Table S11: serum biochemical indexes. [file 9970252.f1.docx]

| Groups | FBW^1^(g) | WGR^2^(%) | SGR^3^(%/d) | FCR^4^ | DFI^5^(%/d) | PER^6^ | LER^7^ | SR^8^(%) |
| --- | --- | --- | --- | --- | --- | --- | --- | --- |
| 50L12 | 68.6400 | 127.2848 | 1.4661 | 1.1768 | 1.6345 | 1.7625 | 7.5335 | 100 |
|  | 64.8800 | 116.5554 | 1.3798 | 1.2960 | 1.7042 | 1.6004 | 6.8405 | 100 |
|  | 67.8400 | 126.7380 | 1.4618 | 1.2037 | 1.6676 | 1.7231 | 7.3648 | 100 |
| P50L9 | 67.3333 | 125.6479 | 1.4532 | 1.2926 | 1.7003 | 1.6084 | 8.7507 | 96 |
|  | 65.1200 | 116.7776 | 1.3816 | 1.2867 | 1.6941 | 1.6157 | 8.7903 | 100 |
|  | 68.7917 | 130.8445 | 1.4939 | 1.2600 | 1.7016 | 1.6499 | 8.9765 | 96 |
| P46L12 | 64.7200 | 115.4461 | 1.3706 | 1.3170 | 1.7213 | 1.6990 | 6.5995 | 100 |
|  | 63.8333 | 112.4945 | 1.3460 | 1.4041 | 1.7154 | 1.5936 | 6.1901 | 96 |
|  | 67.6800 | 125.6000 | 1.4528 | 1.1723 | 1.6150 | 1.9087 | 7.4139 | 100 |
| P46L9 | 65.6667 | 118.8889 | 1.3989 | 1.3823 | 1.7532 | 1.6056 | 8.6883 | 96 |
|  | 66.6250 | 122.6771 | 1.4296 | 1.3382 | 1.7330 | 1.6585 | 8.9745 | 96 |
|  | 64.4400 | 115.0868 | 1.3676 | 1.3246 | 1.7279 | 1.6756 | 9.0670 | 100 |
| P42L12 | 65.6800 | 120.4027 | 1.4112 | 1.2729 | 1.7084 | 1.8861 | 7.0153 | 100 |
|  | 61.6000 | 104.7872 | 1.2800 | 1.3978 | 1.7164 | 1.7175 | 6.3883 | 100 |
|  | 61.9600 | 107.3628 | 1.3023 | 1.3998 | 1.7463 | 1.7151 | 6.3793 | 100 |
| P42L9 | 63.6250 | 110.9582 | 1.3330 | 1.4331 | 1.7345 | 1.6899 | 8.1271 | 96 |
|  | 63.2500 | 111.1148 | 1.3343 | 1.4714 | 1.7825 | 1.6459 | 7.9155 | 96 |
|  | 61.0000 | 103.6048 | 1.2697 | 1.4475 | 1.7641 | 1.6731 | 8.0463 | 100 |

**Table1 Growth performance and feed utilization**

**Table2 Hepatosomatic index(%)**

| Groups | P50L12 | P50L9 | P46L12 | P46L9 | P42L12 | P42L9 |
| --- | --- | --- | --- | --- | --- | --- |
|  | 2.3537 | 2.2236 | 2.6854 | 1.9231 | 2.5349 | 2.1168 |
|  | 2.3133 | 2.3578 | 2.6679 | 2.8226 | 2.5418 | 2.1247 |
|  | 2.6452 | 2.4458 | 2.6547 | 2.6351 | 2.6219 | 2.1169 |
|  | 2.8060 | 2.5135 | 2.5224 | 2.3358 | 2.5522 | 2.8462 |
|  | 2.6042 | 3.0685 | 2.4474 | 2.3216 | 2.6393 | 2.2807 |
|  | 2.9432 | 2.0952 | 2.6296 | 2.3509 | 2.6471 | 2.1842 |
|  | 2.3333 | 2.1167 | 2.5328 | 2.3147 | 2.7656 | 2.1267 |
|  | 2.2766 | 2.5753 | 2.6628 | 2.3219 | 2.6034 | 2.1571 |
|  | 2.7460 | 2.6250 | 3.1613 | 2.7157 | 2.5276 | 2.4211 |
|  | 2.5645 | 2.6349 | 2.5128 | 2.6829 | 2.5234 | 2.1757 |
|  | 2.9967 | 1.9250 | 2.8929 | 2.3023 | 2.7000 | 2.6947 |
|  | 2.8851 | 2.7067 | 2.8421 | 2.4000 | 2.3458 | 2.1216 |
|  | 2.5513 | 2.4189 | 2.5270 | 1.9259 | 2.6885 | 2.2000 |
|  | 2.6486 | 3.0506 | 2.6652 | 2.6667 | 2.6500 | 2.1000 |
|  | 2.6205 | 2.6667 | 2.7631 | 1.9529 | 2.0152 | 1.9500 |
|  | 2.6378 | 2.5598 | 2.7576 | 2.8919 | 2.5870 | 2.2348 |
|  | 2.6297 | 2.5196 | 2.6234 | 2.3462 | 2.5467 | 2.0951 |
|  | 2.9863 | 2.5138 | 2.9540 | 1.9351 | 2.3019 | 2.1032 |
|  | 2.8858 | 2.4169 | 2.9861 | 2.3179 | 2.2133 | 1.9688 |
|  | 2.4458 | 2.5098 | 2.7037 | 2.2236 | 2.0149 | 2.5195 |
|  | 2.4257 | 2.5075 | 2.6278 | 2.3401 | 2.5678 | 2.4444 |
|  | 2.3798 | 3.0667 | 2.6096 | 2.4699 | 2.5789 | 2.0217 |
|  | 2.3894 | 2.4257 | 2.7992 | 1.8148 | 2.5256 | 2.1182 |
|  | 2.7643 | 2.4568 | 2.6774 | 2.3347 | 2.9881 | 2.9710 |
|  | 2.6389 | 2.4326 | 2.5109 | 2.3398 | 2.5359 | 2.1739 |
|  | 2.6657 | 2.4217 | 2.4918 | 1.8072 | 2.6523 | 2.7042 |
|  | 2.6731 | 2.8718 | 2.6789 | 2.6951 | 2.6652 | 2.2279 |
|  | 2.5368 | 2.0779 | 2.6235 | 2.3257 | 2.2879 | 2.2642 |
|  | 2.5982 | 2.4236 | 2.6514 | 2.3218 | 2.5246 | 2.2269 |
|  | 2.7892 | 2.5275 | 2.5739 | 2.3127 | 2.5421 | 2.2564 |

**Table3 Viscerosomatic index(%)**

| Groups | P50L12 | P50L9 | P46L12 | P46L9 | P42L12 | P42L9 |
| --- | --- | --- | --- | --- | --- | --- |
|  | 9.4024 | 9.8333 | 9.0575 | 8.1410 | 10.7794 | 7.5733 |
|  | 10.2603 | 8.8837 | 9.1237 | 8.2317 | 8.9103 | 8.4219 |
|  | 9.1774 | 8.7621 | 8.9873 | 8.4579 | 9.3428 | 8.7143 |
|  | 9.3349 | 9.6622 | 9.2836 | 7.9718 | 9.4521 | 8.5219 |
|  | 9.3789 | 9.7179 | 8.3289 | 8.4819 | 8.4030 | 8.4329 |
|  | 9.4328 | 8.2857 | 8.2778 | 7.9841 | 10.1967 | 9.7101 |
|  | 8.3483 | 9.0411 | 9.2000 | 7.8851 | 9.2941 | 8.3623 |
|  | 10.1932 | 7.5952 | 8.5269 | 9.3049 | 9.5625 | 7.7429 |
|  | 9.7200 | 8.5421 | 9.3459 | 8.8529 | 9.5690 | 8.8732 |
|  | 9.5745 | 8.6621 | 9.3751 | 8.6951 | 9.0000 | 8.8831 |
|  | 9.3762 | 8.2656 | 7.6744 | 7.9070 | 9.5082 | 8.8742 |
|  | 9.3685 | 8.6508 | 7.4717 | 8.1247 | 9.8286 | 8.6527 |
|  | 9.5000 | 8.8400 | 8.3833 | 7.3519 | 9.1781 | 8.3458 |
|  | 9.0964 | 8.5123 | 9.5246 | 8.7451 | 9.2349 | 8.2432 |
|  | 10.1839 | 8.5218 | 9.5000 | 8.1294 | 9.0931 | 8.3426 |
|  | 9.0256 | 9.0000 | 9.4526 | 8.9730 | 9.3125 | 8.7625 |
|  | 9.0946 | 7.3846 | 10.3023 | 8.9359 | 8.9697 | 7.5114 |
|  | 10.1905 | 8.5862 | 9.6186 | 8.3766 | 8.9022 | 8.1333 |
|  | 8.4235 | 8.6412 | 8.6667 | 7.6866 | 9.4000 | 8.2147 |
|  | 9.3569 | 8.6642 | 9.6452 | 9.2143 | 9.7925 | 8.1269 |
|  | 9.3871 | 9.3407 | 9.1667 | 8.7027 | 9.2895 | 9.1077 |
|  | 9.3852 | 7.7403 | 9.1552 | 8.4312 | 9.4179 | 8.1579 |
|  | 9.3881 | 8.5672 | 8.7531 | 8.6129 | 9.0732 | 8.5121 |
|  | 9.9167 | 8.5696 | 9.1944 | 8.3243 | 10.2381 | 8.5123 |
|  | 8.1563 | 8.3108 | 9.1852 | 8.5426 | 8.3768 | 9.0526 |
|  | 9.3798 | 8.7595 | 8.8216 | 8.2281 | 9.5428 | 8.5541 |
|  | 9.4218 | 8.6219 | 10.3827 | 7.2289 | 9.2623 | 9.4316 |
|  | 9.4072 | 8.6421 | 9.1630 | 8.3986 | 7.4815 | 8.6537 |
|  | 9.1746 | 7.5667 | 8.6783 | 8.6535 | 9.2219 | 8.4537 |
|  | 9.1452 | 8.7808 | 9.3217 | 8.5479 | 9.1985 | 8.5133 |

**Table 4 Condition factor (CF, g/cm^3^)**

| Groups | P50L12 | P50L9 | P46L12 | P46L9 | P42L12 | P42L9 |
| --- | --- | --- | --- | --- | --- | --- |
|  | 3.0533 | 3.2799 | 3.1706 | 2.8426 | 2.9566 | 3.1879 |
|  | 3.2472 | 3.1782 | 3.2772 | 2.8220 | 3.1702 | 3.1244 |
|  | 2.7797 | 3.4219 | 3.9229 | 3.1983 | 3.2139 | 3.0611 |
|  | 3.0994 | 3.4126 | 3.2922 | 3.2217 | 3.2087 | 3.9424 |
|  | 3.6005 | 2.9680 | 3.3494 | 3.0755 | 2.7232 | 2.5749 |
|  | 3.2638 | 3.4006 | 2.8919 | 3.6087 | 2.5352 | 3.2494 |
|  | 2.9214 | 3.3043 | 3.4318 | 3.5506 | 2.9264 | 3.0631 |
|  | 3.1709 | 3.6493 | 3.4517 | 3.4692 | 3.4816 | 2.8045 |
|  | 3.0518 | 3.4911 | 3.3176 | 2.7797 | 2.4352 | 2.8045 |
|  | 3.1783 | 3.1469 | 3.4429 | 3.2598 | 2.9696 | 2.7828 |
|  | 3.0483 | 3.6493 | 3.6574 | 3.2111 | 2.7430 | 2.8895 |
|  | 3.2829 | 3.3567 | 3.4954 | 2.8801 | 2.8416 | 2.6437 |
|  | 3.4695 | 3.0729 | 3.1451 | 3.4783 | 3.2608 | 3.2518 |
|  | 3.1406 | 3.5562 | 3.8439 | 3.4596 | 2.9670 | 2.7468 |
|  | 3.4144 | 3.4429 | 3.4721 | 3.1327 | 2.7015 | 3.0231 |
|  | 3.1739 | 3.4318 | 3.4872 | 3.1174 | 2.9087 | 3.3218 |
|  | 3.4496 | 3.4569 | 3.1162 | 3.0755 | 3.5218 | 2.5511 |
|  | 3.0248 | 3.3855 | 3.2724 | 3.4696 | 3.4783 | 3.0755 |
|  | 3.2395 | 3.8079 | 3.3877 | 2.9299 | 3.1994 | 2.8538 |
|  | 2.7241 | 2.8995 | 3.5993 | 3.1211 | 3.1131 | 3.1803 |
|  | 3.1791 | 3.4930 | 3.5880 | 2.9371 | 3.2609 | 3.1392 |
|  | 3.2174 | 3.2317 | 3.8136 | 3.0755 | 2.7798 | 3.1469 |
|  | 3.1494 | 3.4251 | 3.4721 | 3.3868 | 2.9816 | 3.3565 |
|  | 3.1650 | 3.0755 | 2.8995 | 3.2763 | 3.1211 | 2.8763 |
|  | 3.4321 | 3.1238 | 3.4596 | 2.7554 | 3.1127 | 2.8299 |
|  | 3.0983 | 3.0000 | 3.3215 | 3.5493 | 3.1463 | 3.2174 |
|  | 3.3999 | 3.5286 | 3.3359 | 3.3218 | 2.9329 | 2.7893 |
|  | 2.9193 | 3.0723 | 3.1994 | 3.3533 | 3.0215 | 3.0734 |
|  | 3.0734 | 3.4516 | 3.6522 | 2.7312 | 3.0124 | 2.8875 |
|  | 3.1128 | 3.7631 | 3.3368 | 3.0982 | 3.0098 | 3.0419 |

**Table 5 Whole body proximate compositions (%)**

| Groups | Moisture | Crude protein | Crude lipid | Crude ash |
| --- | --- | --- | --- | --- |
| P50L12 | 67.8365 | 16.6027 | 11.5252 | 4.0005 |
|  | 68.6937 | 16.7628 | 11.3722 | 4.0696 |
|  | 69.7308 | 16.2938 | 11.1575 | 4.2330 |
|  |  | 16.5613 | 10.4068 | 4.2986 |
|  |  | 16.7466 | 10.3335 | 4.0650 |
|  |  | 16.8994 | 10.2175 | 3.9754 |
|  |  |  | 9.0781 |  |
|  |  |  | 9.3737 |  |
|  |  |  | 9.0595 |  |
| P50L9 | 70.0494 | 16.8322 | 8.3179 | 4.1576 |
|  | 69.7568 | 17.4901 | 8.5748 | 4.1782 |
|  | 70.3515 | 16.8628 | 8.5621 | 4.3825 |
|  |  | 16.5304 | 8.9567 | 4.2771 |
|  |  | 16.9193 | 8.7742 | 4.0489 |
|  |  | 16.9271 | 8.8773 | 4.3476 |
|  |  |  | 8.4368 |  |
|  |  |  | 8.4336 |  |
|  |  |  | 8.3816 |  |
| P46L12 | 69.0669 | 16.7924 | 9.5625 | 4.1263 |
|  | 70.1712 | 16.9204 | 9.6046 | 4.4478 |
|  | 70.0722 | 16.2863 | 9.7597 | 4.2664 |
|  |  | 16.3900 | 8.5074 | 4.3788 |
|  |  | 17.1287 | 8.5998 | 4.2719 |
|  |  | 17.1500 | 8.4310 | 4.4543 |
|  |  |  | 8.4495 |  |
|  |  |  | 8.2436 |  |
|  |  |  | 8.5290 |  |
| P46L9 | 69.5210 | 16.9245 | 9.0630 | 4.2353 |
|  | 73.9800 | 16.7664 | 8.9926 | 4.2240 |
|  | 70.6422 | 15.0835 | 9.4574 | 3.9659 |
|  |  | 15.2511 | 8.1236 | 3.9289 |
|  |  | 16.8315 | 7.8181 | 4.4054 |
|  |  | 17.0257 | 7.8895 | 4.5223 |
|  |  |  | 8.7629 |  |
|  |  |  | 8.5679 |  |
|  |  |  | 8.3468 |  |
| P42L12 | 70.2065 | 16.5829 | 8.4082 | 4.4039 |
|  | 69.0139 | 16.5377 | 8.6075 | 4.3207 |
|  | 69.3720 | 16.8765 | 8.2345 | 4.5165 |
|  |  | 17.0276 | 9.1652 | 4.4461 |
|  |  | 16.3054 | 9.3306 | 4.1738 |
|  |  | 16.3655 | 9.2743 | 4.2080 |
|  |  |  | 9.9410 |  |
|  |  |  | 9.9845 |  |
|  |  |  | 9.8047 |  |
| P42L9 | 69.4184 | 17.0827 | 8.8284 | 4.2073 |
|  | 70.3208 | 17.2430 | 8.7079 | 4.2258 |
|  | 70.1169 | 16.2000 | 9.1714 | 4.1241 |
|  |  | 16.2071 | 9.0386 | 4.0750 |
|  |  | 17.1447 | 9.1793 | 4.4785 |
|  |  | 17.1291 | 9.0983 | 4.4724 |
|  |  |  | 8.2860 |  |
|  |  |  | 8.1409 |  |
|  |  |  | 8.0666 |  |

**Table 6 Muscle proximate compositions (%)**

| Groups | Moisture | Crude protein | Crude lipid | Crude ash |
| --- | --- | --- | --- | --- |
| P50L12 | 76.7246 | 20.7062 | 2.2295 | 1.3485 |
|  | 76.5142 | 20.5941 | 2.6254 | 1.2806 |
|  | 76.5342 | 20.3797 | 2.4947 | 1.2479 |
|  |  | 20.3876 | 2.5559 | 1.3625 |
|  |  | 20.2670 | 2.6978 | 1.3674 |
|  |  | 20.3047 | 2.4334 | 1.3219 |
|  |  |  | 2.4995 |  |
|  |  |  | 2.5420 |  |
|  |  |  | 2.5447 |  |
| P50L9 | 77.7390 | 19.2908 | 2.2030 | 1.2671 |
|  | 76.8822 | 19.3265 | 2.1535 | 1.2712 |
|  | 77.0883 | 19.7469 | 2.1807 | 1.3840 |
|  |  | 19.9061 | 2.5786 | 1.3680 |
|  |  | 19.4846 | 2.7856 | 1.1964 |
|  |  | 19.5612 | 2.5421 | 1.1793 |
|  |  |  | 2.7405 |  |
|  |  |  | 2.6830 |  |
|  |  |  | 2.6627 |  |
| P46L12 | 77.8098 | 19.3150 | 1.9450 | 1.2780 |
|  | 77.0739 | 19.3791 | 2.0104 | 1.2769 |
|  | 76.5013 | 18.9551 | 1.8144 | 1.1090 |
|  |  | 19.2120 | 3.0278 | 1.0635 |
|  |  | 19.3955 | 3.0164 | 1.1174 |
|  |  | 19.5283 | 3.1207 | 1.1555 |
|  |  |  | 3.2431 |  |
|  |  |  | 3.2821 |  |
|  |  |  | 3.2601 |  |
| P46L9 | 79.0260 | 18.2708 | 2.4921 | 1.1042 |
|  | 79.3313 | 18.3313 | 2.2327 | 1.1088 |
|  | 78.4473 | 18.1852 | 2.2282 | 1.0448 |
|  |  | 18.2047 | 1.7282 | 1.1002 |
|  |  | 18.8477 | 2.0608 | 1.2527 |
|  |  | 18.9673 | 1.8925 | 1.2851 |
|  |  |  | 2.1332 |  |
|  |  |  | 1.9562 |  |
|  |  |  | 1.9686 |  |
| P42L12 | 78.8556 | 18.5736 | 2.4283 | 1.1154 |
|  | 78.0867 | 18.6406 | 2.2716 | 1.1030 |
|  | 77.4427 | 18.6543 | 2.0599 | 1.1176 |
|  |  | 18.4893 | 2.8855 | 1.1159 |
|  |  | 19.2052 | 2.8071 | 1.1239 |
|  |  | 19.4312 | 2.8511 | 1.0800 |
|  |  |  | 2.6911 |  |
|  |  |  | 2.6289 |  |
|  |  |  | 2.4788 |  |
| P42L9 | 78.0676 | 19.0580 | 2.3302 | 1.1827 |
|  | 78.2525 | 18.5569 | 2.2275 | 1.1483 |
|  | 79.5707 | 18.8865 | 2.1764 | 1.2649 |
|  |  | 18.8763 | 1.7430 | 1.1221 |
|  |  | 18.7642 | 1.7942 | 1.1101 |
|  |  | 18.8398 | 1.8523 | 1.1017 |
|  |  |  | 1.9468 |  |
|  |  |  | 1.8431 |  |
|  |  |  | 1.9081 |  |

**Table 7 Liver proximate compositions (%)**

| Groups | Moisture | Crude protein | Crude lipid | Crude ash |
| --- | --- | --- | --- | --- |
| P50L12 | 65.2456 | 11.3217 | 14.2371 | 0.9845 |
|  | 66.5639 | 10.2637 | 15.6029 | 1.0237 |
|  | 66.7507 | 11.3748 | 16.2798 | 1.1415 |
|  |  | 10.5879 | 15.2244 | 1.0129 |
|  |  | 10.3271 | 14.8951 | 1.01289 |
|  |  | 11.2791 | 14.9875 | 0.9785 |
|  |  |  | 16.2789 |  |
|  |  |  | 15.8975 |  |
|  |  |  | 16.2579 |  |
| P50L9 | 68.0298 | 10.8967 | 12.3789 | 1.1126 |
|  | 67.8637 | 10.2793 | 12.7795 | 1.1257 |
|  | 66.3571 | 11.2375 | 12.7698 | 1.0789 |
|  |  | 10.8109 | 11.6789 | 1.1178 |
|  |  | 10.3746 | 12.6575 | 1.1289 |
|  |  | 11.2759 | 11.2479 | 1.9845 |
|  |  |  | 12.6578 |  |
|  |  |  | 11.9578 |  |
|  |  |  | 12.1249 |  |
| P46L12 | 68.0155 | 10.8314 | 15.2637 | 0.9545 |
|  | 67.2708 | 10.7846 | 15.2678 | 0.9956 |
|  | 68.5679 | 10.2719 | 15.2672 | 1.0212 |
|  |  | 10.8174 | 16.2317 | 1.1268 |
|  |  | 10.9753 | 14.2978 | 1.0236 |
|  |  | 9.8471 | 15.2971 | 0.9971 |
|  |  |  | 16.4562 |  |
|  |  |  | 15.6789 |  |
|  |  |  | 15.2698 |  |
| P46L9 | 69.3556 | 9.9745 | 10.3789 | 1.2256 |
|  | 69.1237 | 10.2978 | 9.12046 | 1.3478 |
|  | 70.6422 | 10.9875 | 9.4573 | 1.0459 |
|  |  | 10.5627 | 9.1567 | 1.0978 |
|  |  | 10.2418 | 9.8571 | 1.1648 |
|  |  | 11.3538 | 9.1458 | 1.0679 |
|  |  |  | 9.5216 |  |
|  |  |  | 9.5317 |  |
|  |  |  | 9.5216 |  |
| P42L12 | 70.8537 | 11.2316 | 15.1523 | 0.9916 |
|  | 68.2079 | 10.9872 | 15.6892 | 1.0125 |
|  | 70.7539 | 10.3789 | 14.6895 | 1.1123 |
|  |  | 11.2378 | 14.5895 | 0.9875 |
|  |  | 11.3715 | 14.3389 | 1.1024 |
|  |  | 11.3455 | 14.2227 | 1.2312 |
|  |  |  | 15.9878 |  |
|  |  |  | 15.6678 |  |
|  |  |  | 14.6553 |  |
| P42L9 | 70.3657 | 10.2479 | 7.2367 | 1.231 |
|  | 69.5171 | 9.5347 | 8.6425 | 1.223 |
|  | 70.2349 | 10.3175 | 8.9212 | 1.3312 |
|  |  | 11.0795 | 7.9512 | 1.3302 |
|  |  | 11.2397 | 8.1268 | 1.2289 |
|  |  | 10.6978 | 8.8951 | 1.2479 |
|  |  |  | 7.9521 |  |
|  |  |  | 8.0526 |  |
|  |  |  | 8.9452 |  |

**Table 8 Essential amino acid composition of muscle(%)**

| Groups | Threonine | Valine | Methionine | Isoleucine | Leucine | Phenylalanine | Lysine | Histidine | Arginine | Total |
| --- | --- | --- | --- | --- | --- | --- | --- | --- | --- | --- |
| P50L12 | 4.212 | 3.733 | 1.365 | 3.651 | 7.4 | 3.679 | 7.522 | 2.256 | 5.037 | 38.855 |
|  | 4.287 | 3.881 | 1.67 | 3.85 | 7.523 | 3.636 | 7.664 | 2.304 | 5.148 | 39.963 |
|  | 4.167 | 3.833 | 2.081 | 3.759 | 7.307 | 3.616 | 7.618 | 2.346 | 5.047 | 39.774 |
|  | 4.242 | 3.997 | 2.143 | 3.953 | 7.306 | 3.599 | 7.478 | 2.266 | 5.134 | 40.118 |
|  | 4.237 | 3.957 | 2.222 | 3.867 | 7.358 | 3.609 | 7.49 | 2.258 | 5.13 | 40.128 |
|  | 4.085 | 3.523 | 1.911 | 3.428 | 7.109 | 3.463 | 7.26 | 2.249 | 4.947 | 37.975 |
| P50L9 | 4.189 | 3.984 | 0.799 | 3.596 | 6.999 | 4.038 | 7.899 | 1.574 | 5.121 | 38.199 |
|  | 4.137 | 3.905 | 0.912 | 3.579 | 6.889 | 3.906 | 7.811 | 1.723 | 5.07 | 37.932 |
|  | 4.176 | 3.736 | 0.954 | 3.281 | 6.978 | 4.062 | 7.825 | 1.510 | 5.122 | 37.644 |
|  | 4.168 | 3.874 | 0.897 | 3.505 | 6.945 | 4.009 | 7.854 | 1.538 | 5.112 | 37.902 |
|  | 3.853 | 3.497 | 1.11 | 3.086 | 6.445 | 3.777 | 7.256 | 1.594 | 4.671 | 35.289 |
|  | 3.937 | 3.679 | 0.795 | 3.219 | 6.583 | 3.945 | 7.401 | 1.463 | 4.831 | 35.853 |
| P46L12 | 4.109 | 3.877 | 1.187 | 3.598 | 6.861 | 3.859 | 7.895 | 1.411 | 5.137 | 37.934 |
|  | 4.09 | 3.726 | 1.2 | 3.247 | 6.825 | 3.984 | 7.656 | 1.587 | 4.997 | 37.312 |
|  | 3.885 | 3.562 | 0.907 | 3.186 | 6.489 | 3.766 | 7.308 | 1.386 | 4.787 | 35.276 |
|  | 4.121 | 3.873 | 1.291 | 3.453 | 6.761 | 3.879 | 7.637 | 2.476 | 4.966 | 38.457 |
|  | 3.965 | 3.539 | 0.783 | 3.147 | 6.541 | 3.78 | 7.331 | 1.37 | 4.887 | 35.343 |
|  | 3.917 | 3.566 | 0.889 | 3.062 | 6.517 | 3.89 | 7.336 | 1.416 | 4.804 | 35.397 |
| P46L9 | 4.244 | 3.689 | 1.388 | 3.194 | 6.936 | 3.893 | 7.751 | 2.834 | 5.074 | 39.003 |
|  | 4.413 | 3.937 | 1.131 | 3.546 | 7.347 | 4.153 | 8.272 | 1.491 | 5.492 | 39.782 |
|  | 4.011 | 3.637 | 1.693 | 3.206 | 6.637 | 3.795 | 7.437 | 1.565 | 4.887 | 36.868 |
|  | 4.804 | 4.444 | 0.758 | 4.038 | 7.895 | 4.484 | 8.799 | 1.685 | 6.008 | 42.915 |
|  | 4.42 | 3.987 | 1.511 | 3.566 | 7.31 | 4.228 | 8.257 | 1.53 | 5.436 | 40.245 |
|  | 4.838 | 4.13 | 1.154 | 3.775 | 7.876 | 4.28 | 8.858 | 1.855 | 5.96 | 42.726 |
| P42L12 | 4.277 | 3.835 | 0.967 | 3.451 | 7.116 | 4.102 | 8.141 | 1.43 | 5.288 | 38.607 |
|  | 4.382 | 3.82 | 1.258 | 3.455 | 7.257 | 4.01 | 8.164 | 1.606 | 5.416 | 39.368 |
|  | 4.628 | 4.383 | 1.127 | 4.092 | 7.723 | 4.409 | 8.729 | 1.574 | 5.773 | 42.438 |
|  | 4.284 | 4.005 | 1.345 | 3.731 | 7.159 | 4.153 | 8.149 | 1.433 | 5.306 | 39.565 |
|  | 4.729 | 4.379 | 0 | 3.851 | 7.82 | 4.681 | 8.921 | 1.948 | 5.713 | 42.042 |
|  | 4.799 | 4.303 | 1.116 | 3.836 | 7.915 | 4.546 | 8.978 | 1.837 | 5.859 | 43.189 |
| P42L9 | 3.874 | 3.752 | 0.698 | 3.317 | 6.511 | 3.999 | 7.523 | 1.331 | 4.744 | 35.749 |
|  | 3.799 | 3.444 | 1.058 | 3.07 | 6.292 | 3.731 | 7.108 | 1.39 | 4.592 | 34.484 |
|  | 4.158 | 3.823 | 1.063 | 3.509 | 6.86 | 3.932 | 7.764 | 1.507 | 5.111 | 37.727 |
|  | 3.992 | 3.67 | 1.292 | 3.266 | 6.567 | 3.9 | 7.456 | 1.475 | 4.848 | 36.466 |
|  | 4.147 | 3.942 | 0.731 | 3.511 | 6.846 | 4.072 | 7.782 | 1.597 | 5.036 | 37.664 |
|  | 4.026 | 3.596 | 0.892 | 3.173 | 6.594 | 3.835 | 7.399 | 1.424 | 4.84 | 35.779 |

**Table 9 Non-essential amino acid composition of muscle(%)**

| Groups | Proline | Tyrosine | Serine | Glutamic acid | Glycine | Alanine | Cysteine | Aspartic acid | Total |
| --- | --- | --- | --- | --- | --- | --- | --- | --- | --- |
| P50L12 | 2.769 | 3.358 | 4.005 | 13.483 | 4.639 | 6.098 | 1.882 | 9.478 | 45.712 |
|  | 2.81 | 3.279 | 4.051 | 13.644 | 4.649 | 6.166 | 1.759 | 9.602 | 45.96 |
|  | 2.688 | 3.26 | 3.936 | 13.009 | 4.472 | 5.934 | 1.899 | 9.329 | 44.527 |
|  | 2.761 | 3.285 | 4.145 | 13.004 | 4.91 | 6.122 | 1.847 | 9.404 | 45.478 |
|  | 2.944 | 3.151 | 4.044 | 13.126 | 4.711 | 6.004 | 1.859 | 9.417 | 45.256 |
|  | 2.801 | 2.913 | 3.91 | 12.843 | 4.482 | 5.89 | 1.774 | 9.238 | 43.851 |
| P50L9 | 2.617 | 3.421 | 3.939 | 13.113 | 4.214 | 5.655 | 1.883 | 9.411 | 44.253 |
|  | 2.601 | 3.268 | 3.859 | 12.857 | 4.201 | 5.606 | 1.77 | 9.278 | 43.44 |
|  | 2.693 | 3.413 | 3.978 | 13.045 | 4.346 | 5.713 | 2.006 | 9.500 | 44.695 |
|  | 2.564 | 3.364 | 3.937 | 12.98 | 4.286 | 5.641 | 1.869 | 9.415 | 44.056 |
|  | 2.554 | 3.35 | 3.654 | 12.021 | 3.93 | 5.286 | 1.847 | 8.796 | 41.438 |
|  | 2.487 | 3.418 | 3.727 | 12.252 | 3.975 | 5.3 | 1.944 | 8.864 | 41.967 |
| P46L12 | 2.628 | 3.276 | 3.813 | 12.829 | 4.202 | 5.596 | 1.663 | 9.317 | 43.324 |
|  | 2.464 | 3.115 | 3.857 | 12.776 | 4.111 | 5.521 | 1.99 | 9.271 | 43.105 |
|  | 2.552 | 3.232 | 3.693 | 12.136 | 3.988 | 5.291 | 1.814 | 8.75 | 41.456 |
|  | 2.669 | 3.218 | 3.936 | 12.671 | 4.348 | 5.635 | 1.825 | 9.218 | 43.52 |
|  | 2.509 | 3.288 | 3.793 | 12.317 | 4.163 | 5.393 | 1.756 | 8.942 | 42.161 |
|  | 2.615 | 3.309 | 3.727 | 12.262 | 4.166 | 5.352 | 1.939 | 8.93 | 42.3 |
| P46L9 | 2.612 | 3.435 | 4.127 | 13.389 | 4.481 | 5.873 | 1.87 | 9.711 | 45.498 |
|  | 2.792 | 3.631 | 4.208 | 13.973 | 4.632 | 6.033 | 1.968 | 10.045 | 47.282 |
|  | 2.59 | 3.332 | 3.829 | 12.518 | 4.182 | 5.444 | 1.841 | 9.103 | 42.839 |
|  | 3.17 | 3.694 | 4.58 | 14.953 | 4.871 | 6.37 | 2.172 | 10.652 | 50.462 |
|  | 2.787 | 3.534 | 4.2 | 13.794 | 4.632 | 6.021 | 1.965 | 10.065 | 46.998 |
|  | 3.134 | 3.443 | 4.618 | 15.142 | 5.068 | 6.57 | 1.855 | 11.023 | 50.853 |
| P42L12 | 2.707 | 3.573 | 4.081 | 13.833 | 4.499 | 5.838 | 1.931 | 9.747 | 46.209 |
|  | 2.843 | 3.478 | 4.241 | 13.917 | 4.655 | 6.06 | 1.784 | 10.063 | 47.041 |
|  | 2.865 | 3.708 | 4.361 | 14.574 | 4.816 | 6.293 | 1.835 | 10.522 | 48.974 |
|  | 2.614 | 3.48 | 4.051 | 13.453 | 4.429 | 5.808 | 1.847 | 9.707 | 45.389 |
|  | 3.016 | 3.885 | 4.521 | 15.235 | 4.776 | 6.394 | 2.123 | 10.739 | 50.689 |
|  | 2.965 | 3.777 | 4.643 | 15.117 | 4.924 | 6.573 | 2.037 | 10.958 | 50.994 |
| P42L9 | 2.437 | 3.339 | 3.691 | 12.602 | 3.953 | 5.272 | 1.911 | 8.798 | 42.003 |
|  | 2.405 | 3.028 | 3.587 | 12.171 | 3.805 | 5.141 | 1.745 | 8.644 | 40.526 |
|  | 2.587 | 3.289 | 3.927 | 13.027 | 4.211 | 5.616 | 1.751 | 9.396 | 43.804 |
|  | 2.432 | 3.225 | 3.805 | 12.503 | 4.003 | 5.349 | 1.8 | 8.982 | 42.099 |
|  | 2.602 | 3.482 | 3.948 | 12.95 | 4.225 | 5.531 | 1.835 | 9.265 | 43.838 |
|  | 2.495 | 3.396 | 3.892 | 12.923 | 4.063 | 5.396 | 1.848 | 9.106 | 43.119 |

**Table 10 Major fatty acids of muscle**

| Groups | C14:0 | C16:0 | C16:1 | C18:0 | C18:1n-7 | C18:1n-9 | C18:2n-6 | C20:1 | C20:5n-3 | C22:1n-9 | 22:6n-3 |
| --- | --- | --- | --- | --- | --- | --- | --- | --- | --- | --- | --- |
| P50L12 | 3.2528 | 17.8578 | 5.483 | 4.1162 | 3.8954 | 17.4536 | 5.4534 | 2.3848 | 7.7238 | 2.5266 | 14.9292 |
|  | 3.1292 | 17.0177 | 5.0598 | 3.7143 | 3.704 | 16.708 | 5.1841 | 2.3268 | 7.391 | 2.4317 | 14.2101 |
|  | 3.4622 | 18.5011 | 5.8986 | 4.0554 | 3.9276 | 18.3958 | 5.3623 | 2.4577 | 7.3088 | 2.6444 | 12.8529 |
|  | 3.4608 | 18.2765 | 5.8768 | 4.0405 | 3.8631 | 18.5117 | 5.3301 | 2.5113 | 7.3408 | 2.7271 | 12.4923 |
|  | 3.5246 | 17.9195 | 5.7791 | 3.9523 | 3.8883 | 17.9427 | 5.4334 | 2.5663 | 7.5569 | 3.2274 | 13.3193 |
|  | 3.5431 | 17.8907 | 5.7268 | 3.9787 | 3.858 | 18.0511 | 5.4602 | 2.5901 | 7.5948 | 3.21 | 13.2169 |
| P50L9 | 3.3815 | 18.3238 | 6.0585 | 3.903 | 3.9479 | 19.6061 | 6.0502 | 2.2435 | 7.2549 | 2.4247 | 12.1067 |
|  | 3.3714 | 18.3361 | 6.0032 | 3.9209 | 4.0519 | 19.6002 | 6.0264 | 2.2864 | 7.1588 | 2.4619 | 12.0924 |
|  | 3.3503 | 18.1119 | 5.9233 | 4.0029 | 4.0049 | 19.4802 | 6.2277 | 2.2883 | 7.2959 | 2.4131 | 12.0898 |
|  | 3.4022 | 18.1655 | 5.968 | 4.0035 | 4.0589 | 19.4182 | 6.2453 | 2.2576 | 7.3316 | 2.41 | 11.9635 |
|  | 3.432 | 18.1099 | 6.039 | 3.9426 | 3.9915 | 19.1991 | 6.175 | 2.3497 | 7.3096 | 3.0173 | 11.9382 |
|  | 3.4399 | 17.9565 | 6.0652 | 3.8942 | 3.9914 | 19.0431 | 6.2149 | 2.3502 | 7.4114 | 2.5966 | 12.0929 |
| P46L12 | 3.4481 | 18.0819 | 5.495 | 4.2111 | 3.8072 | 17.3057 | 5.5341 | 2.5168 | 7.4426 | 2.7607 | 13.8526 |
|  | 3.4818 | 18.2612 | 5.591 | 4.227 | 3.8607 | 17.227 | 5.5136 | 2.5209 | 7.4165 | 2.7134 | 13.9319 |
|  | 3.7777 | 17.724 | 5.8931 | 3.8147 | 3.8141 | 17.5579 | 5.5554 | 2.7421 | 7.5987 | 3.4961 | 12.9013 |
|  | 3.9434 | 18.0166 | 6.0652 | 3.7853 | 3.7246 | 17.6978 | 5.5737 | 3.7253 | 7.4764 | 3.4156 | 12.4828 |
|  | 3.8956 | 18.4454 | 6.2458 | 3.9655 | 3.9642 | 18.2865 | 5.7158 | 2.5781 | 7.1816 | 3.2291 | 11.3233 |
|  | 3.8197 | 18.3691 | 6.1262 | 3.994 | 3.8604 | 18.4471 | 5.6503 | 2.6498 | 7.24 | 3.3559 | 11.5221 |
| P46L9 | 3.3681 | 18.1969 | 5.8287 | 4.2265 | 3.8071 | 18.4428 | 6.1027 | 2.3808 | 7.1214 | 2.5946 | 12.2867 |
|  | 3.3979 | 18.4425 | 5.7475 | 4.3455 | 4.0179 | 18.7153 | 6.1442 | 2.4473 | 7.052 | 2.6755 | 12.0708 |
|  | 3.3776 | 18.5393 | 5.7545 | 4.3236 | 3.9858 | 18.3617 | 6.0687 | 2.3685 | 7.0837 | 2.6178 | 12.4428 |
|  | 3.3195 | 18.2004 | 5.4497 | 4.2904 | 3.9476 | 18.2272 | 6.0492 | 2.3628 | 7.1539 | 2.6414 | 12.49 |
|  | 3.2302 | 18.069 | 5.365 | 4.3163 | 3.8889 | 17.9419 | 6.3423 | 2.3054 | 7.4622 | 2.4262 | 13.4138 |
|  | 3.2406 | 18.0458 | 5.3984 | 4.1964 | 3.8205 | 17.8177 | 6.3397 | 2.2957 | 7.5303 | 2.3924 | 13.4943 |
| P42L12 | 3.4634 | 17.7482 | 4.8904 | 4.2988 | 3.6592 | 16.1504 | 5.5817 | 3.6355 | 7.6257 | 3.2312 | 14.8487 |
|  | 3.4627 | 17.7426 | 4.8838 | 4.3463 | 3.7274 | 16.1248 | 5.5805 | 2.6607 | 7.5669 | 3.2774 | 14.5275 |
|  | 3.6963 | 17.5513 | 5.5404 | 4.236 | 3.7826 | 16.6427 | 5.662 | 2.6919 | 7.6942 | 3.4174 | 13.4228 |
|  | 3.7272 | 17.5644 | 5.461 | 4.1988 | 3.7793 | 16.8048 | 5.706 | 2.7177 | 7.5944 | 3.4005 | 13.4888 |
|  | 3.8194 | 18.3004 | 5.5812 | 4.467 | 3.849 | 17.1701 | 5.5835 | 2.7702 | 7.1804 | 3.4768 | 12.5764 |
|  | 3.8907 | 18.7879 | 5.3919 | 4.608 | 3.9672 | 17.4811 | 5.4776 | 2.8429 | 6.7306 | 3.6392 | 11.6626 |
| P42L9 | 3.3165 | 17.8197 | 5.4863 | 4.2051 | 3.9603 | 17.9903 | 6.1961 | 2.3577 | 7.4842 | 2.4746 | 13.5607 |
|  | 3.309 | 17.8478 | 5.3164 | 4.2291 | 3.9183 | 18.0418 | 6.1575 | 2.4007 | 7.3323 | 2.5232 | 13.4957 |
|  | 3.1842 | 18.1059 | 5.4249 | 4.2252 | 3.9332 | 17.7568 | 6.0987 | 2.3149 | 7.366 | 2.4358 | 13.828 |
|  | 3.2279 | 18.4257 | 5.4048 | 4.2985 | 3.9615 | 18.0133 | 6.0969 | 2.3426 | 7.2314 | 2.4704 | 13.493 |
|  | 3.2453 | 17.891 | 5.2744 | 4.2547 | 3.9341 | 17.6456 | 6.2385 | 2.3895 | 7.3407 | 2.5275 | 14.0562 |
|  | 3.256 | 17.8196 | 5.3312 | 4.2159 | 3.9009 | 17.4559 | 6.2368 | 2.3596 | 7.426 | 2.4595 | 13.9924 |

**Table 11 Serum biochemical indexes**

| Groups | TP(g/L) | ALB(g/L) | TG(mmol/L) | CHO(mmol/L) | HDL(mmol/L) | LDL(mmol/L) | ALT（U/L） | AST（U/L） |
| --- | --- | --- | --- | --- | --- | --- | --- | --- |
| P50L12 | 35.33 | 22.1897 | 2.5718 | 4.9995 | 3.8247 | 0.6547 | 30.0092 | 34.9229 |
|  | 33.2579 | 23.1791 | 2.6554 | 5.2051 | 3.6871 | 0.6678 | 26.2250 | 36.5695 |
|  | 32.0139 | 27.5146 | 2.5911 | 5.1950 | 3.5248 | 0.6547 | 32.6791 | 37.3480 |
|  | 30.4498 | 22.1572 | 2.2378 | 4.9617 | 3.6789 | 0.6878 | 30.9926 | 36.5734 |
|  | 38.1647 | 23.1567 | 2.7964 | 4.8617 | 3.1478 | 0.6178 | 27.0574 | 32.5290 |
|  | 33.1576 | 25.1671 | 2.6876 | 5.2802 | 3.5478 | 0.6678 | 31.2814 | 36.5734 |
|  | 35.6179 | 24.6879 | 2.5053 | 4.9681 | 3.6178 | 0.6789 | 27.1260 | 34.1260 |
|  | 38.1129 | 24.6178 | 2.5816 | 4.9879 | 3.6784 | 0.6788 | 30.5439 | 38.3708 |
|  | 35.6679 | 26.3178 | 2.7966 | 5.0728 | 3.6789 | 0.6789 | 31.2814 | 35.2292 |
| P50L9 | 36.7781 | 25.4587 | 2.2885 | 4.3844 | 3.4561 | 0.5578 | 30.4868 | 32.4868 |
|  | 36.7798 | 26.31782 | 2.1809 | 4.6906 | 3.1478 | 0.5678 | 28.2671 | 36.7077 |
|  | 36.4451 | 26.1478 | 2.5099 | 4.7118 | 3.2178 | 0.5947 | 30.9551 | 39.0229 |
|  | 36.1879 | 24.3417 | 2.2632 | 4.9705 | 3.1297 | 0.6048 | 32.4914 | 32.4914 |
|  | 39.2457 | 22.3178 | 2.2764 | 4.9732 | 3.3127 | 0.6178 | 29.5039 | 29.5039 |
|  | 38.2479 | 23.0489 | 2.2628 | 4.9714 | 3.4578 | 0.6278 | 26.1591 | 36.1591 |
|  | 36.1279 | 24.6517 | 2.6358 | 4.9732 | 3.4781 | 0.6237 | 26.2850 | 36.2850 |
|  | 35.6172 | 25.6178 | 2.4657 | 4.6104 | 3.7841 | 0.6317 | 25.9827 | 35.9827 |
|  | 34.1287 | 24.1247 | 2.6319 | 4.8058 | 3.1247 | 0.6278 | 30.7509 | 32.7509 |
| P46L12 | 31.4781 | 22.1674 | 2.5025 | 4.8633 | 3.4178 | 0.6314 | 25.2591 | 35.1776 |
|  | 34.1297 | 24.1687 | 2.3929 | 4.5551 | 3.1247 | 0.6348 | 30.4123 | 34.9229 |
|  | 35.1467 | 28.1674 | 2.4025 | 4.8642 | 3.1278 | 0.6678 | 30.9551 | 32.5290 |
|  | 30.1971 | 23.1578 | 2.0929 | 4.8033 | 3.2256 | 0.6957 | 28.5061 | 34.9229 |
|  | 34.1862 | 24.1367 | 2.0571 | 4.7917 | 3.2478 | 0.6347 | 30.5680 | 30.5680 |
|  | 38.7489 | 22.3571 | 2.1711 | 4.9054 | 3.3378 | 0.6457 | 32.0050 | 32.0050 |
|  | 36.1827 | 25.6417 | 2.3085 | 4.7446 | 3.1178 | 0.6549 | 27.9159 | 30.5553 |
|  | 33.1279 | 23.7891 | 2.3443 | 4.8065 | 3.6478 | 0.6378 | 29.9758 | 36.6458 |
|  | 34.18921 | 24.6148 | 2.5234 | 4.5260 | 3.1475 | 0.6817 | 31.5820 | 38.7257 |
| P46L9 | 34.6175 | 27.2157 | 2.1101 | 4.4373 | 3.1271 | 0.5517 | 34.4875 | 37.5243 |
|  | 36.1298 | 23.6178 | 1.9697 | 4.1795 | 3.1278 | 0.5479 | 29.2779 | 34.4931 |
|  | 32.97418 | 26.1673 | 1.9055 | 4.1788 | 3.1782 | 0.5317 | 30.2642 | 34.4931 |
|  | 38.2176 | 26.3178 | 1.8046 | 4.1016 | 3.3317 | 0.5217 | 27.9159 | 31.8424 |
|  | 37.5126 | 23.6179 | 1.7353 | 4.1122 | 3.0127 | 0.5214 | 27.9159 | 31.8424 |
|  | 37.31298 | 27.65147 | 2.0746 | 4.2364 | 3.2147 | 0.5519 | 26.3523 | 29.3265 |
|  | 37.8156 | 27.6547 | 2.0407 | 4.4385 | 3.0124 | 0.5317 | 29.4875 | 35.0194 |
|  | 33.1267 | 23.3671 | 2.1707 | 4.0568 | 3.0217 | 0.5417 | 31.2957 | 31.8405 |
|  | 33.1274 | 24.6178 | 2.1904 | 4.0557 | 2.978 | 0.5617 | 29.7700 | 37.3480 |
| P42L12 | 38.6789 | 26.3179 | 2.1923 | 4.6928 | 2.7745 | 0.5961 | 29.9551 | 37.2850 |
|  | 32.1678 | 22.3157 | 2.2865 | 4.7302 | 2.6684 | 0.5816 | 29.8346 | 39.9912 |
|  | 36.9845 | 26.3187 | 2.3185 | 4.6966 | 2.7489 | 0.5719 | 26.7614 | 37.4084 |
|  | 31.5748 | 26.2157 | 2.5525 | 4.9633 | 2.5478 | 0.5843 | 29.9912 | 29.9912 |
|  | 36.5471 | 24.62178 | 2.3062 | 4.6957 | 2.5671 | 0.5749 | 25.9827 | 35.9827 |
|  | 36.5418 | 26.3478 | 2.1143 | 4.7911 | 2.6478 | 0.5919 | 27.3480 | 37.3480 |
|  | 36.841 | 26.3178 | 1.9955 | 4.6959 | 2.6479 | 0.5517 | 26.0227 | 32.5290 |
|  | 36.5871 | 26.3187 | 2.2650 | 4.3065 | 2.6479 | 0.5749 | 28.3005 | 36.5695 |
|  | 35.1825 | 22.3154 | 2.3385 | 4.5942 | 2.6679 | 0.5749 | 27.8499 | 34.3915 |
| P42L9 | 36.8718 | 23.6217 | 2.0757 | 4.6335 | 2.1785 | 0.4479 | 29.1138 | 35.3870 |
|  | 39.1654 | 25.6217 | 1.8642 | 3.9764 | 2.1278 | 0.4465 | 29.7700 | 33.0515 |
|  | 32.5784 | 23.6174 | 1.9579 | 4.6163 | 2.3178 | 0.4578 | 26.8518 | 33.9305 |
|  | 33.65718 | 22.6219 | 1.8774 | 4.3772 | 2.4795 | 0.4795 | 25.4058 | 32.4922 |
|  | 31.2674 | 21.3647 | 1.7564 | 3.7746 | 2.6784 | 0.4879 | 26.0000 | 31.5243 |
|  | 33.1567 | 26.6257 | 1.8995 | 3.9441 | 2.3784 | 0.4679 | 28.2349 | 33.7001 |
|  | 36.6478 | 25.6179 | 1.8850 | 3.9254 | 2.127 | 0.4762 | 29.5430 | 30.8694 |
|  | 36.647 | 23.6175 | 2.0789 | 4.0462 | 2.3378 | 0.4841 | 29.1138 | 35.1175 |
|  | 33.1589 | 23.6148 | 1.6578 | 4.1189 | 2.3675 | 0.4796 | 31.8932 | 33.3013 |
